# Recent Secondary Contacts, Linked Selection, and Variable Recombination Rates Shape Genomic Diversity in the Model Species *Anolis carolinensis*

**DOI:** 10.1093/gbe/evz110

**Published:** 2019-05-27

**Authors:** Yann Bourgeois, Robert P Ruggiero, Joseph D Manthey, Stéphane Boissinot

**Affiliations:** 1 New York University Abu Dhabi, United Arab Emirates; 2 Department of Biological Sciences, Texas Tech University

**Keywords:** *Anolis carolinensis*, recombination, divergence, selection

## Abstract

Gaining a better understanding on how selection and neutral processes affect genomic diversity is essential to gain better insights into the mechanisms driving adaptation and speciation. However, the evolutionary processes affecting variation at a genomic scale have not been investigated in most vertebrate lineages. Here, we present the first population genomics survey using whole genome resequencing in the green anole (*Anolis carolinensis*). Anoles have been intensively studied to understand mechanisms underlying adaptation and speciation. The green anole in particular is an important model to study genome evolution. We quantified how demography, recombination, and selection have led to the current genetic diversity of the green anole by using whole-genome resequencing of five genetic clusters covering the entire species range. The differentiation of green anole’s populations is consistent with a northward expansion from South Florida followed by genetic isolation and subsequent gene flow among adjacent genetic clusters. Dispersal out-of-Florida was accompanied by a drastic population bottleneck followed by a rapid population expansion. This event was accompanied by male-biased dispersal and/or selective sweeps on the X chromosome. We show that the interaction between linked selection and recombination is the main contributor to the genomic landscape of differentiation in the anole genome.

## Introduction

Nucleotide variation along a DNA sequence results from the interactions between multiple processes that either generate new alleles (e.g., recombination, mutation) or affect the fate of these alleles in populations (e.g., selection, demography, and speciation). The variable outcome of these interactions along the genome can result in heterogeneous patterns of diversity and divergence at both intra- and interspecific scales (Begun and Aquadro 1992; Nachman and Payseur 2012; Cruickshank and Hahn 2014; Roux et al. 2014; Seehausen et al. 2014; Wolf and Ellegren 2017). Given their importance in divergence and speciation, quantifying these processes has been at the core of evolutionary genomics for the last decade. With the advent of next-generation sequencing and the continuous development of novel analytical tools, it has become possible to properly quantify the impact of recombination (Booker et al. 2017; Kawakami et al. 2017), selection (Begun and Aquadro 1992; Barrett et al. 2008; Mullen and Hoekstra 2008; Cai et al. 2009), and demographic history (Gutenkunst et al. 2009; Excoffier et al. 2013; Roux et al. 2016) on diversity patterns in several vertebrates. Ultimately, such investigations have the power to answer outstanding biological questions such as the role of sex chromosomes, the nature of reproductive barriers, or the timing of gene flow and selection during differentiation (Wolf and Ellegren 2017). Assessing the effects of these mechanisms in a range of organisms is crucial to inform current debates about their relative importance (Bierne et al. 2011; Kern and Hahn 2018; Pouyet et al. 2018; Jensen et al. 2019) with a broader set of empirical data.

Thorough analyses of the factors affecting genome diversity at the peri-specific level have been performed in a small number of vertebrate species (see Ellegren et al. 2012; Sousa et al. 2013; Poelstra et al. 2014; Booker et al. 2017; Han et al. 2017; Kawakami et al. 2017) but several major clades of vertebrates have not been investigated at all. Among those are the nonavian reptiles, a speciose group of vertebrates that harbor a wide diversity of morphology and adaptation. Anoles in particular have been abundantly studied to understand the mechanisms underlying adaptation. This neotropical group of squamates diversified during the Cenozoic, and constitute a model system for understanding speciation and adaptation in ectotherms (Losos et al. 2004; Glor et al. 2005; Losos 2009; Kolbe et al. 2017; Lapiedra et al. 2018). The analysis of anoles genomes has also provided considerable insights on genome evolution in vertebrates (Alföldi et al. 2011; Fujita et al. 2011; Tollis and Boissinot 2011, 2013; Figuet et al. 2015; Costantini et al. 2016; Ruggiero et al. 2017). At last, they display a dramatic physiological, morphological, and behavioral diversity (Lailvaux et al. 2004; Glor et al. 2005; Wade 2012; Campbell-Staton et al. 2018; Lapiedra et al. 2018). Given this importance as a model species, we decided to perform a study on genome-wide variation in the green anole (*Anolis carolinensis*) to better understand its microevolutionary dynamics, expanding previous genetic, and genomic work (Tollis et al. 2012; Tollis and Boissinot 2014; Manthey et al. 2016).

The green anole is the first nonavian reptile for which the whole genome was sequenced (Alföldi et al. 2011) and its population structure is relatively well known (Tollis et al. 2012; Tollis and Boissinot 2014; Campbell-Staton et al. 2016; Manthey et al. 2016; Ruggiero et al. 2017). Whole-genome resequencing of green anoles populations would be an opportunity to better understand the drivers and constraints that act on their radiation at a resolution that was not allowed by the previous genetic data sets.

The green anole colonized Florida from Cuba (Glor et al. 2005; Campbell-Staton et al. 2012; Tollis et al. 2012; Tollis and Boissinot 2014; Manthey et al. 2016) between 6 and 12 Ma and diversified into five genetic groups: South Florida (SF), Eastern Florida (EF), Western Florida (WF), Gulf Atlantic (GA), and Carolinas (CA). Populations in Florida likely diverged in allopatry on island refugia before coming back into contact due to sea-level oscillations during the Pleistocene. Colonization of the rest of North America seems to be more recent, with two clades having probably expanded in the last 500,000 years (Manthey et al. 2016). It is the only species in the *Anolis* genus to have colonized temperate climates without human intervention.

Here, we present results obtained from whole-genome resequencing of five genetic clusters of the green anole. We provide a detailed assessment of the multiple factors that are likely to impact the green anole’s genetic diversity at a genome-wide scale. We demonstrate that the combined effects of regional variation in recombination rate, linked selection, and migration are responsible for the heterogeneous genomic landscape of diversity and divergence in the green anole.

## Materials and Methods

### DNA Extraction and Whole Genome Sequencing

Whole genome sequencing libraries were generated from *A.carolinensis* liver tissue samples collected between 2009 and 2011 (Tollis et al. 2012), and *Anolisporcatus* and *Anolisallisoni* tissue samples generously provided by Breda Zimkus from the Museum of Comparative Zoology at Harvard University. For each of the 29 samples, DNA was isolated from ethanol preserved tissue using Ampure beads per the manufacturers’ protocol. Illumina TRU-Seq paired end libraries were generated using 200 ng of DNA per sample and sequenced at the NYUAD Center for Genomics And Systems Biology Sequencing Core (http://nyuad.nyu.edu/en/research/infrastructure-and-support/core-technology-platforms.html; Last accessed on June 7, 2019) with an Illumina HiSeq 2500. Read quality was assessed with FastQCv0.11.5 (http://www.bioinformatics.babraham.ac.uk/projects/fastqc; Last accessed on June 7, 2019) and Trimmomatic (Bolger et al. 2014) was subsequently used to remove low quality bases, sequencing adapter contamination and systematic base calling errors. Specifically, the parameters “trimmomatic_adapter.fa: 2:30:10 TRAILING:3 LEADING:3 SLIDINGWINDOW:4:15 MINLEN:36” were used. Samples had an average of 1,519,339,234 read pairs, and after quality trimming 93.3% were retained as paired reads and 6.3% were retained as single reads. Sequencing data from this study have been submitted to the Sequencing Read Archive (https://www.ncbi.nlm.nih.gov/sra; last accessed: June 7, 2019) under the BioProject designation PRJNA533001.

### Sequence Alignment and SNP Calling

Quality trimmed reads were aligned to the May 2010 assembly of the *A. carolinensis* reference genome (Broad AnoCar2.0/anoCar2; GCA_000090745.1; Alföldi et al. 2011) and processed for SNP detection with the assistance of the NYUAD Bioinformatics Core, using NYUAD variant calling pipeline (last accessed in June 2017). Briefly, the quality-trimmed FastQ reads of each sample were aligned to the AnoCar2.0 genome using the BWA-mem short read alignment approach (Li and Durbin 2011) and resulting SAM files were converted into BAM format, sorted, and indexed using SAMtools (Li et al. 2009). Picard was then used to identify insertions, deletions, and duplications in the sorted BAM files (http://broadinstitute.github.io/picard/; Last accessed on June 7, 2019) and evaluated using SAMtools (stats and depth). Alignments contained an average of 204,459,544 reads that passed QC, 97.75% mapping and 91.93% properly paired (supplementary table S1, Supplementary Material online). Each individual resequenced genome was then processed with GATK for indel realignment, SNP and indel discovery, and genotyping, following GATK Best Practices (Depristo et al. 2011; Van Der Auwera et al. 2014). GATK joint genotyping was conducted with HaplotypeCaller for increased sensitivity and confidence, and results were selectively compared with results generated from SAMtools mpileup (Li et al. 2009). Filtering was performed in VCFtools (Danecek et al. 2011), with the following criteria: a 6× minimum depth of coverage per individual, a 15× maximum average depth of coverage, no more than 40% missing data across all 29 samples, a minimum quality score of 20 per site, and a minimum genotype quality score of 20.

### Population Structure

To assess genetic structure, we conducted a clustering analysis using discriminant analysis of principal components (DAPC) on a subset of ∼6,500 SNPs with <20% missing data and randomly thinned every 10 kb to minimize linkage disequilibrium (LD) between markers while retaining enough variants for inference. DAPC (Jombart et al. 2010) first estimates principal components (PC) describing variance in SNP data sets, then performs a discriminant analysis on these PC axes to identify genetic groupings. We selected the clustering model with the highest support using the Bayesian Information Criterion (BIC). We retained two principal components that explained ∼40% of the total variance, and two of the linear discriminants. The probability for each individual to be assigned to a specific cluster was summarized by a barplot with the function compoplot() provided with the DAPC R package. We also described relationships between individuals with the same data set using the network algorithm implemented in Splitstree v4 (Huson and Bryant 2006). Lastly, we filtered the entire SNP data set to include one million randomly sampled SNPs present in a minimum of 80% of the individuals for use as input in RAxML v8 (Stamatakis 2014). We used RAxML to create a maximum-likelihood phylogeny, using the GTRGAMMA model of sequence evolution, and 100 rapid bootstraps to assess support for the phylogeny with the highest likelihood.

We further examined patterns of diversity and the shape of the allele frequency spectrum in each cluster by computing two summary statistics, the average number of pairwise differences *θ*_π_ (or nucleotide diversity) per bp, and Tajima’s *D*, for nonoverlapping 5-kb windows using the software POPGENOME (Pfeifer et al. 2014). We removed windows overlapping ambiguities in the green anole genome using BEDTOOLS v2.25.0 (Quinlan and Hall 2010).

### Demographic Estimates without Gene Flow

We used the multiepoch model implemented in SMC++ (Terhorst et al. 2017) to reconstruct population size trajectories and time since population split for each of the five genetic clusters of green anoles. This software is an extension of the Pairwise Sequentially Markov Coalescent (Li and Durbin 2011) that uses the spatial arrangement of polymorphisms along genome sequences to naively infer variation in effective population sizes and splitting time between populations. It has the advantage of using both information related to the site frequency spectrum and patterns of LD to make demographic inferences. Another benefit of this algorithm is that it is phase-insensitive, limiting the propagation of phasing errors that can bias effective population size estimates for recent times (Terhorst et al. 2017).

Within each of the five genetic clusters, we created one data set per individual for each of the six autosomes and combined those individual data sets to reconstruct past variation in effective population sizes. A mutation rate of 2.1×10^−10^ per site per generation and a generation time of 1 year (Tollis and Boissinot 2014) were assumed to translate coalescence times into years. We set a polarization error of 0.5 since the ancestral allele could not be determined for many loci. We also estimated splitting times between genetic clusters. However, these estimates should be taken with caution as the method assumes that no gene flow occurs after the split.

### Effective Sex-Ratio

Sex-biased contribution to the gene pool is a critical aspect of demographic dynamics and is often impacted by variation in social structure between populations. We used the algorithm implemented in KIMTREE (Gautier and Vitalis 2013; Clemente et al. 2018) to estimate branch lengths from our SNP data set and infer the effective sex-ratios (ESR) for each of the five genetic clusters. This method is robust to LD, small sample sizes, and demographic events such as bottlenecks and expansions. To increase the number of usable markers, and since the authors recommend working with recently diverged populations, we focused on the recent northward colonization, and included individuals from the East Florida, Gulf Atlantic, and Carolinas genetic clusters.

Briefly, the method builds a hierarchical Bayesian model to estimate the evolution of SNP frequencies along branches of a population tree provided by the user. Genetic drift along branches is estimated by a time-dependent diffusion approximation. In this framework, branch length τ is proportional to the time since divergence in generations (*t*) scaled by the effective population size (*N*_e_), such that *τ* ≡ *t*/2*N*_e_. The method can jointly contrast allele frequencies between autosomal and sex-linked markers to estimate the relative contribution of males and females to each generation (ESR). The ESR can then be seen as a comparison of the effective population sizes estimates obtained from autosomes and the X chromosome.

We sexed individuals by taking advantage of the expected relationship between depths of coverage at autosomal and sex-linked loci in males and females. Since females are XX and males XY, the latter are expected to display a two-times lower coverage at X-linked sites compared with autosomal loci (supplementary fig. 1, Supplementary Material online). We then adjusted allele frequencies for all X-linked scaffolds, including Linkage Group b (Alföldi et al. 2011) and several scaffolds (GL343282, GL343364, GL343550, GL343423, GL343913, GL343947, GL343338, GL343417) recently identified as belonging to the green anole’s sex chromosome (Rupp et al. 2017). We counted one haplotype per male and two per female. To obtain confidence intervals over ESR estimates, we generated 50 pseudoreplicated data sets by randomly sampling 5,000 autosomal and 5,000 sex-linked SNPs with no missing data. The algorithm was started with 25 pilot runs of 1,000 iterations each to adjust the parameters of the Monte Carlo Markov Chain (MCMC). The MCMC itself was run for 100,000 generations and sampled every 25 iterations after a burn-in of 50,000 iterations. Convergence for all parameters was assessed by visually inspecting posterior sampling in R (R Core team 2016). For each replicate *i*, we estimated the support for biased sex-ratio (*S_i_*) such as:

$$S_{i}=\;1\;–\;2\;\left|p_{i}–\;0.5\;\right|.$$

with *S_i_* <0.05 being interpreted as a strong support for biased sex-ratio and where *p_i_* is the proportion of posterior MCMC samples with an ESR >0.5.

### Model Comparison of Demographic Scenarios

None of the previous population genetics studies of green anoles has ever precisely quantified the strength nor the timing of gene flow between genetic clusters. We addressed this issue by comparing different demographic scenarios for two pairs of sister clades (EF and GA; EF and WF) that included the most individuals (at least 11).We used the diffusion approximation-based likelihood approach implemented in the ∂a∂i software (Gutenkunst et al. 2009). We compared a set of scenarios of strict isolation (SI), isolation with migration (IM), ancient migration (AM) with one or two (PAM) periods of gene flow and secondary contact (SC) with one or two (PSC) periods of gene flow (see Christe et al. 2017 for a detailed summary). We added complexity to this set of basic scenarios by allowing for a combination of population expansion (prefix “ex”), heterogeneous asymmetric migration rates (suffix “2M2P”) and heterogeneous effective population size (suffix “2N”) among loci. These additions were made to incorporate the genome-wide effects of selection on linked neutral sites (so-called “linked selection”) and model genomic islands resisting gene flow (Cruickshank and Hahn 2014). We also tested scenarios with both asymmetric migration rates and heterogeneous population sizes but were unable to reach convergence. Overall, we compared 34 scenarios combining these features, using a set of scripts available on dryad (Christe et al. 2017) and a modified version of ∂a∂i (v1.7.0) kindly provided by Christelle Fraïsse (available at http://methodspopgen.com/wp-content/uploads/2017/12/dadi-1.7.0_modif.zip; Last accessed on June 7, 2019). We extracted for each pairwise comparison a set of ∼12,000 SNPs with no missing data and thinned every 100,000 bp to meet the requirement of independence among loci that is needed to properly compare the composite likelihoods estimated by ∂a∂i. We extracted the unfolded joint sites frequency spectra (SFS) by polarizing alleles using *A. porcatus* and *A. allisoni* as references. We considered ancestral the allele found at a minimal frequency of 75% in those two individuals or found fixed in one of them if the other individual was missing. We note that the ∂a∂i models include a parameter (O) estimating the proportion of correctly polarized sites. We evaluated each model 30 times and retained the replicate with the highest likelihood for model comparison. Models were compared using the Akaike information criterion (AIC). For the best model, we calculated uncertainties over the estimated parameters using a nonparametric bootstrap procedure, creating 100 pseudo-observed data sets by resampling with replacement from the SFS. We used the procedure implemented in the dadi.Godambe.GIM_uncert() script to obtain a maximum-likelihood estimate of 95% confidence intervals (Coffman et al. 2016). ∂a∂i parameters are scaled by the ancestral population size *N*_ref_. For the sake of comparison with SMC++ estimates, parameters were converted into demographic units by estimating the ancestral effective population size as the harmonic mean of the SMC++ estimates before splitting time for all pairs of populations.

### Estimating Recombination Rates

We used the LDHat software (McVean et al. 2002) to estimate effective recombination rates (*ρ *= 4 *N*_e_*r* with *r* the recombination rate per generation and *N* the effective population size) along the green anole genome. This method has been successfully used to obtain recombination maps for data sets similar to ours in terms of sequencing depth and sample sizes (Auton et al. 2012). Unphased genotypes were converted into LDHat format using VCFtools (option –ldhat). Since LDHat assumes that samples are drawn from a panmictic population, we focused on the Eastern Florida clade for which sampling effort was the highest (*n* = 8 diploid individuals). We used precomputed likelihood lookup tables with an effective population mutation rate (*θ*) of 0.001, which was the closest from the *θ* value estimated from our data set (*θ* ∼ 0.004) and used the lkgen module to generate a table fitting the number of observed samples (16 chromosomes). Recombination rates were estimated over 500-kb windows with 100-kb overlaps using the Bayesian reversible MCMC scheme implemented in the interval module. The chain was run for 1,000,000 iterations and sampled every 5,000 iterations with a large block penalty of 20 to avoid overfitting and minimize random noise. The first 100,000 generations were discarded as burn-in. Convergence under these parameters was confirmed by visually inspecting MCMC traces for a subset of windows. We averaged *ρ* estimates over nonoverlapping 100-kb windows, or over coding sequences for subsequent analyses.

### Summary Statistics for Differentiation and LD

To assess whether the joint effects of selection and low recombination on diversity and differentiation, we computed two measures of divergence (*F*_ST_ and *d*_XY_) over nonoverlapping 100-kb windows for the three divergent Floridian lineages. These lineages were chosen because of their relative demographic stability (see Results). We picked 100,000 bp to reduce spatial autocorrelation between statistics of adjacent windows, since no further substantial LD decay could be observed over this distance for the two populations with the largest sample sizes (supplementary fig. 2, Supplementary Material online), pairwise LD (measured as *r*^2^) was computed using VCFtools (Danecek et al. 2011). Comparison between those two statistics for a given genomic region has been proposed as a way to disentangle the effects of gene flow and selection (Cruickshank and Hahn 2014). For the sake of simplicity, correlations between differentiation statistics and recombination were estimated using a Spearman’s correlation test in R, although we note that measurements cannot be fully considered independent. As a sanity check, we computed the *ZZ* statistics (Rozas et al. 2001) to assess whether LDHat estimates of *ρ* were consistent with the genomic distribution of LD. This statistic contrasts LD between adjacent pairs of SNPs to LD calculated over all pairwise comparisons in a given window. High values are suggestive of increased intragenic recombination. We also computed the average frequency of polymorphic derived alleles (average DAF) in the EF cluster since it included the most individuals, using *A. porcatus* and *A. allisoni* to polarize alleles, and excluding sites with at least six individuals genotyped over eight. DAF has been recently used to estimate the effects of linked selection in humans, an excess of ancestral alleles being expected in regions under the influence of background selection (Pouyet et al. 2018).

Because *ρ* is the effective recombination rate and depends on the effective population size, it is directly correlated to local reduction of diversity due to linked selection. Thus, a low value of *ρ* may be observed in regions linked to selection even if *r* itself is not significantly different from the rest of the genome. To reduce this correlation, we calculated *ρ/θ*_π_, with *θ*_π_ the nucleotide diversity computed in POPGENOME. Since *θ*_π_ is an estimator of 4*N*µ, with µ the mutation rate, this statistic represents the ratio between *r* and the mutation rate µ (see Wang et al. 2016 for an example).

We examined the average DAF across five quantiles of *ρ/θ*_π_ to assess whether lower rates of recombination were associated with changes in the frequency of derived alleles that may be due to linked selection. We also compared the average DAF between the genomic background and regions of high relative and low absolute divergence which may be candidates for stronger linked selection. These regions were defined as regions belonging both to the top 20% quantile for *F*_ST_ and the lowest 20% quantile for *d*_XY._

## Results

### Statistics for Whole Genome Resequencing

Twenty-seven green anoles (*A.carolinensis*) sampled across the species’ range and covering the five genetic clusters identified in previous analyses (Tollis and Boissinot 2014; Manthey et al. 2016) were chosen for whole-genome resequencing. We also included two samples from the closely related species *A. porcatus* and *A. allisoni* as outgroups. Sequencing depth was between 7.22× and 16.74×, with an average depth of 11.45× (supplementary table S1, Supplementary Material online). About 74,920,333 variants with <40% missing data were retained after the first round of filtering (Materials and Methods).

### Population Structure and Nucleotide Variation Reveal a Reduced Diversity in Northern Populations

**Fig. 1.**
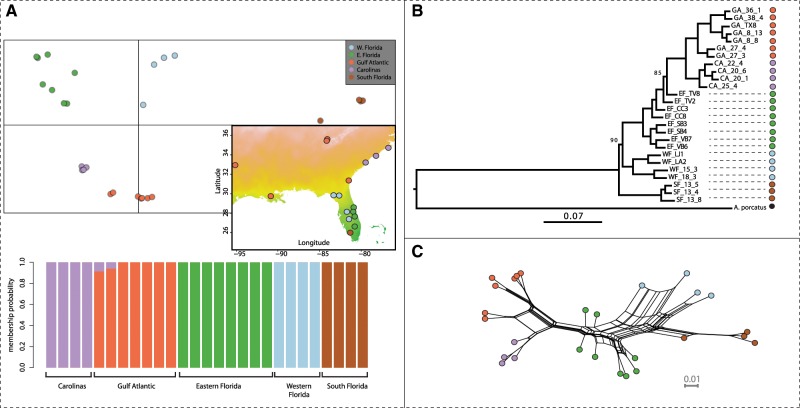
—Genetic structure in *Anolis carolinensis* from whole-genome SNP data. (*A*) Results from the DAPC analysis highlighting the five clusters inferred from the analysis of ∼6,500 SNPs thinned every 10 kb and with <20% missing data. The map reports the coordinates of the localities used in this study and the genetic clusters they belong to. (*B*) RAxML phylogeny based on one million SNPs randomly sampled across the genome. All 100 bootstrap replicates supported the reported topology, except for two nodes with support of 90 and 85. One individual from South Florida was removed due to a high rate of missing data. (*C*) Network representation of the relatedness between samples as inferred by Splitstree v4. Color codes match those in parts (*A*) and (*B*).

We first assessed geographic structure across the distribution of *A. carolinensis*. To this end, we used >6,500 SNPs thinned every 10 kb and with <20% missing data and identified k = 5 as the most likely number of genetic clusters with DAPC (fig. 1*A* and supplementary fig. 3, Supplementary Material online). Three groups were identified within Florida, while individuals from the rest of North-America were assigned to two clusters. These groups were consistent with the clusters identified in previous genetic studies (Tollis et al. 2012; Tollis and Boissinot 2014; Manthey et al. 2016). Possible introgression from Carolinas was observed for two Gulf Atlantic individuals (fig. 1*A*). A maximum-likelihood phylogeny estimated in RAxML based on one million random SNPs and a network analysis of relatedness in Splitstree further supported this clustering (fig. 1*B* and *C*). Results closely matched previous findings, with South Florida (SF) being the sister clade of all other groups. The two northernmost clusters, Gulf Atlantic (GA) and Carolinas (CA), clustered together in the RaxML phylogeny. Eastern Florida (EF) constituted a paraphyletic group in the phylogeny in which GA and CA were nested. This is likely due to incomplete lineage sorting induced by the high and constant effective population sizes of populations from Florida (see below), or to ongoing or recent gene flow resulting in the inclusion of loci with different coalescence times. At last, the Western Florida (WF) cluster was basal to all other groups except South Florida (SF).

Nucleotide diversity was the lowest in GA and CA (table 1) despite the large geographic area covered by these two genetic clusters. Average Tajima’s *D* values ranged between −0.8 (WF) and 0.14 (GA). Positive Tajima’s *D* values suggest recent population contraction, while negative Tajima’s *D* are expected in the case of recent population expansion (Tajima 1989), although both population substructure and linked selection may impact it. Northern clusters (CA and GA) displayed the highest average Tajima’s *D*, possibly due to reductions in population sizes and relaxation of linked selection (see below).

**Table 1:** Diversity and Tajima’s *D* (±SD) for Each of the Five Genetic Clusters, Averaged over Nonoverlapping 5-kb Windows across the Genome

Statistics	CA	GA	EF	WF	SF
Nucleotide diversity	0.00155±0.00154	0.00177±0.00153	0.00330±0.0021	0.00341±0.0022	0.00279±0.002
Tajima’s *D*	−0.17±1.49	0.14±0.0015	−0.73±0.002	−0.80±0.0022	−0.66±0.002

### Recent Population Expansion and Male-Biased Sex-Ratios in Northern Populations

**Fig. 2.**
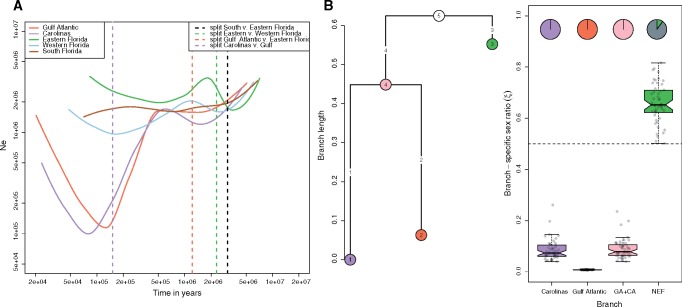
—Variation in effective population sizes with time and comparison of drift between autosomes and sex-linked scaffolds. (*A*) Reconstruction of past variations in effective population sizes (*N*_e_) inferred by SMC++. Dashed vertical lines correspond to the estimated splitting times between the five genetic clusters previously inferred. We assume a mutation rate of 2.1×10^−10^/bp per generation and a generation time of 1 year. (*B*) Average branch lengths obtained from autosomal data and ESRs (*ξ*) inferred from KIMTREE. A set of 5,000 autosomal and 5,000 sex-linked markers were randomly sampled to create 50 pseudoreplicated data sets on which the analysis was run. The analysis was run on the three most closely related populations. Pie charts indicate the proportion of replicates for which we observed significant support (*S_i_*<0.01) in favor of a biased sex-ratio.

We then reconstructed the demographic history of each genetic cluster. To this end, we used the whole set of filtered SNPs with <40% missing data to infer past changes in effective population sizes (*N*_e_) without any a priori demographic model with SMC++ (fig. 2*A*). All populations from Florida showed rather stable demographic trajectories, with some evidence for population expansion in EF and WF. Assuming a mutation rate of 2.1×10^−10^/bp per year (Tollis and Boissinot 2014), present population sizes were in the range of 500,000 to 5,000,000 individuals for each population, in accordance with previous analyses based on target capture markers (Manthey et al. 2016). Northern populations (CA and GA) showed a clear signature of expansion starting between 200,000 and 100,000 years ago, following a bottleneck that started between 500,000 and 1,000,000 years in the past. We also estimated the splitting times between the different groups but since this model assumes no gene flow after the split, the estimates are likely to be biased toward the present. The split between GA and CA occurred shortly before these populations expanded, in accordance with the previously proposed hypothesis of double colonization following the Gulf and Atlantic coasts (Tollis and Boissinot 2014). In Florida, divergence events took place between 3 and 2 Ma. The relative order of splitting events was consistent with the topology obtained from our phylogeny and previous studies.

Since anoles exhibit sex differences in dispersal (Johansson et al. 2008), we tested whether the recent colonization of new and possibly suboptimal habitats could lead to unequal contribution of males and females to the gene pool or selection at sex-linked loci (fig. 2*B*). We built a population tree and quantified genetic signatures of biased sex-ratio with the algorithm implemented in KIMTREE. We focused on the three populations that diverged most recently, GA, CA, and EF. Note that the length of branch *i* (*τ_i_*) represents time in generations (*t_i_*) scaled by the effective population size for this branch such as *τ_i_*_=_*t_i_*/2*N*_e,__*i*_ (Clemente et al. 2018). Branch lengths were particularly high for the CA and GA lineages compared with EF, as expected in the case of stronger drift (fig. 2*B*). This is in line with their smaller effective population sizes and the bottleneck inferred by SMC++. We found evidence for a strongly male-biased ESR in CA and GA, but not in EF which was slightly female-biased. Indeed, nucleotide diversity was substantially more reduced at sex-linked scaffolds in GA than in EF when compared with autosomal diversity (supplementary fig. 4, Supplementary Material online). Note that sex-ratios are the proportion of females effectively contributing to the gene pool along each branch of the tree and should not be interpreted directly in terms of census size. The GA cluster displayed the strongest bias, with an estimated ratio of less than one female for 100 males, suggesting strong sex-bias in the founding population or strong male-biased dispersal during population expansion. The CA cluster and the inner branch leading to CA and GA showed a ratio of approximately 10 females for 100 males. All 50 replicates displayed a high support for a male-biased sex-ratio in CA and GA, while only five replicates supported a female-biased sex-ratio in EF (i.e., the Markov chain almost systematically explored sex-ratios >0.5 in only five replicates).

### Gene Flow at SC Has Homogenized Green Anole Populations

**Fig. 3.**
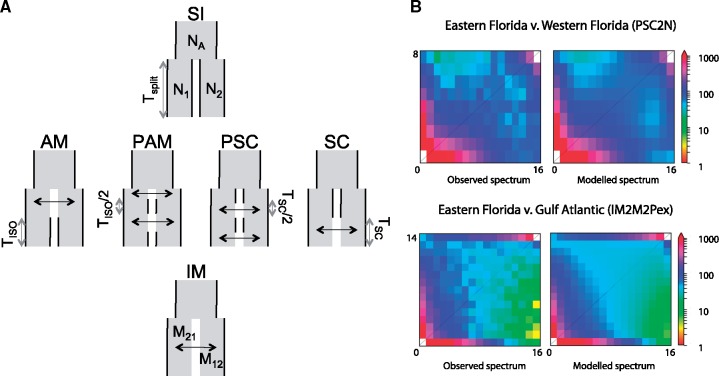
—(*A*) Graphic description of the six categories of ∂a∂i models tested over pairs of green anole genetic clusters. Each model describes a scenario where two populations diverge from an ancestral one, with varying timing and strength of gene flow after their split. SI, strict isolation; AM, ancestral migration, where populations first exchange gene flow then stops *T*_iso_ generations ago; PAM, ancestral migration with two periods of contact lasting *T*_iso_/2 generations; SC, secondary contact where populations still exchange gene flow at present time; PSC, secondary contact with two periods of contact lasting *T*_sc_/2 generations; IM, isolation with constant migration and no interruption of gene flow. Models were constrained so that *T*_iso_ and *T*_sc_ lasted at least ∼50,000 years. Reproduced with the authorization of Christelle Fraïsse. (*B*) Fitting of the best models for the EF (*N* = 16) versus GA (*N* = 14) and EF versus WF (*N* = 8) comparisons. Both models fit the observed data sets as indicated by the similar spectra between observation and simulation. The “2 *N*” suffix means that background selection was added to the base model by modeling heterogeneous effective population sizes across loci. The “2M2P” suffix means that heterogeneity in gene flow was incorporated into the model. The “ex” suffix means that exponential population size change was introduced in the base model.

We tested whether gene flow and its interruption may have played a role in shaping the genomic landscape of differentiation in green anoles (fig. 3). We focused on two pairs of genetic clusters. The first comparison was between the EF and WF clusters, which are two populations with high and stable population sizes (according to SMC++), that both live in subtropical Florida. This comparison should be suited to detect the long-term effects of interrupted gene flow since there is already evidence that these clades may have been isolated by sea rising during interglacial periods (Tollis and Boissinot 2014; Manthey et al. 2016). In addition, since populations from Florida have remained relatively stable over the last 2 Myr, our power to detect the expected correlations between recombination and diversity should be enhanced in the case of linked selection (Burri 2017; Torres et al. 2019). The second comparison was between the EF and GA clusters, the latter corresponding to a recent expansion northward and adaptation to temperate environments.

We ran ∂a∂i models to assess the effects of differential gene flow and linked selection. We compared a set of 34 divergence scenarios, allowing gene flow and effective population sizes to vary with time and across loci. Briefly, heterogeneity in gene flow (suffix 2M2P) was implemented by dividing the site frequency spectrum into three sets of loci with proportions 1-*P*_1_-*P*_2_, *P*_1_, and *P*_2_. Assuming that the first population is WF and the second EF, the first set of loci (1-*P*_WF_-*P*_EF_) is modeled with all parameters from the base model. The two other sets are modeled with no gene flow toward WF (*P*_WF_) or EF (*P*_EF_) and represent genomic islands resisting gene flow in WF and EF, respectively. To simulate the reduction in diversity expected under purifying selection at linked, nonrecombinant (*nr*) sites, two sets of loci were modeled at frequencies 1−*nr* and *nr* (suffix 2 *N*). The first set was modeled with all parameters from the base model, the other with the same parameters but with effective population sizes reduced by a background selection factor (*bf*).

Strict-isolation models (SI) consistently displayed the lowest likelihood and highest AIC, clearly supporting a role for gene flow in homogenizing green anoles genomes. For the comparison between EF and WF, models including heterogeneous population sizes performed better than models with heterogeneous gene flow. Among scenarios with gene flow, SC with one and two periods of gene flow (SC and PSC) often received the highest support (fig. 3*B* and supplementary fig. 5, Supplementary Material online). Parameters estimated from the best models are shown in table 2. There was no substantial gain in likelihood when adding expansion to scenario of two SCs with background selection (PSC2N), and models with heterogeneous migration displayed lower likelihood. The PSC2N model supported a scenario where about *nr* = 65% of the genome was affected by selection at linked sites, suggesting a rather large effect of low recombination and purifying selection on diversity. These Eastern and Western Floridian genetic clusters experienced long periods of isolation lasting ∼2 Myr, followed by periods of SC lasting ∼125,000 years in total.

**Table 2:** Summary of Best-Supported Demographic Models

Comparison	Model	Na12	Na1	Na2	N1	N2	m2->1	m1->2	Tiso	Tsc	Tscg	*P*_1_	*P*_2_	*nr*	*bf*	O	logLikelihood	AIC
GA vs. EF	IM2M2Pex	2,156,641	3,538,759	2,438,866	371,048	7,132,328	2.42E-07	2.57E-07	NA	521,532*	1,615,603	0.83	0.95	NA	NA	0.97	−1,045.99	2,113.97
GA vs. EF	IMex	2,156,641	5,971,380	2,137,291	364,329	6,755,470	1.93E-07	2.36E-07	NA	61,217**	1,962,652	NA	NA	NA	NA	0.97	−1,048.14	2,114.29
GA vs. EF	SC2M2Pex	2,156,641	4,214,885	2,296,060	367,662	7,049,892	2.12E-07	2.58E-07	139,258**	229,611*	1,723,615	0.91	0.95	NA	NA	0.97	−1,046.01	2,116.01
GA vs. EF	PSCex	2,156,641	4,126,463	2,402,541	367,459	6,926,709	1.93E-07	2.35E-07	58,466**	110,560	1,713,849	NA	NA	NA	NA	0.97	−1,048.03	2,116.06
GA vs. EF	SCex	2,156,641	4,150,102	2,359,593	369,470	6,934,978	1.91E-07	2.36E-07	97,752**	249,043*	1,719,867	NA	NA	NA	NA	0.97	−1,048.05	2,116.11
GA vs. EF	PSC2M2Pex	2,156,641	3,873,718	2,394,203	370,665	7,005,838	2.04E-07	2.58E-07	79,128**	124,565*	1,672,998	0.93	0.95	NA	NA	0.97	−1,046.12	2,116.24
GA vs. EF	IM2Nex	2,156,641*	3,106,584*	2,766,204	360,422	6,961,498	1.99E-07	2.34E-07	NA	615,678	1,461,807	NA	NA	0.60**	0.81*	0.97	−1,048.09	2,118.17
EF vs. WF	PSC2N	2,091,300	NA	NA	5,181,876	5,389,218	1.94E-06	6.93E-07	1,064,768	759,07	NA	NA	NA	0.60	0.25	0.98	−669.91	1,357.81
EF vs. WF	SC2N	2,091,305	NA	NA	5,252,609*	5,330,317*	1.18E-06	4.25E-07	1,952,690	144,821*	NA	NA	NA	0.57	0.26	0.98	−676.66	1,371.33

Note.—PSC2N, secondary contact with two periods in isolation and heterogeneous effective population sizes across the genome; SCex, secondary contact with an episode of population expansion following secondary contact; SC2M2P and IM2M2P, models of secondary contact and constant gene flow with heterogeneous migration rates along the genome. The ancestral size (*N*_a12_) before the split was calculated from the SMC++ output to facilitate comparisons. For the -ex models, following the initial split, populations have a population size of *N*_1a_ and *N*_2a_ followed by an exponential growth that leads to their current sizes *N*_1_ and *N*_2_. In basic models, populations have constant sizes *N*_1_ and *N*_2_ since the split. *nr*, proportion of the genome displaying an effective population size of *bf* times the population size displayed by the remaining 1−*nr* fraction not affected by linked selection; *O*, proportion of sites for which the ancestral state was correctly inferred; *P*_1_ and *P*_2_, the proportion of sites resisting gene flow in populations 1 and 2; *T*_iso_, total time spent in isolation. For the PSC model, populations are isolated twice in their history for *T*_iso_/2 generations and are connected twice for *T*_sc_/2 generations (see fig. 3*A*). *T*_sc_, time during which stable populations stay connected; *T*_scg_, time since population size change (with gene flow). The total time during which populations were connected is *T*_scg_+*T*_sc_. For each model, the set of best estimates is shown. Uncertainties over parameters were measured by SDs obtained from 100 bootstrap replicates. No star, uncertainties are below ±20% of point estimates; *, uncertainties between ±20% and ±50%; **, uncertainties between ±50% and ±150%. Cells with “NA” values correspond to parameters that were not part of a given model.

For the comparison between GA and EF, we confirmed the decrease in effective population size detected by SMC++ in GA compared with EF, with a present effective population size 20 times lower in GA than in EF. We note that unlike SMC++, ∂a∂i was not able to detect the recent rebound in size following the bottleneck, even after allowing for more past changes in effective population sizes (data not shown). The model with the smallest AIC was the IM2M2Pex model, followed by models of SC (PSCex, SCex, and SC2M2Pex). We therefore present results obtained for several representative models (table 2). All models supported a scenario with extensive gene flow, with high uncertainties for the time spent in isolation for SC models. Models with the highest likelihood and lowest AIC incorporated genomic barriers to gene flow in GA, with ∼10–20% of loci resisting introgression from Florida and ∼5% resisting gene flow from GA.

### Recombination and Linked Selection Shape Genome Differentiation and Diversity

It has been suggested that SCs can lead to the emergence of genomic islands resisting gene flow, that display higher differentiation than regions that have been homogenized (Payseur et al. 2004; Teeter et al. 2008; Larson et al. 2014; Malinsky et al. 2015; McGee et al. 2016). The diversity of such islands may also be higher, as they diverged and accumulated mutations before gene flow resumed. On the other hand, selection at linked sites can also generate genomic islands, as it reduces diversity and lead to an increase of relative measures of differentiation (Noor and Bennett 2009; Cruickshank and Hahn 2014; Burri et al. 2015). Some of the best supported models in ∂a∂i suggested a widespread impact of selection in Florida, reducing diversity at linked sites over ∼60% of the genome. We therefore tested the role of low recombination in shaping the genomic landscape of diversity and differentiation in green anoles in a context of SC.

**Fig. 4.**
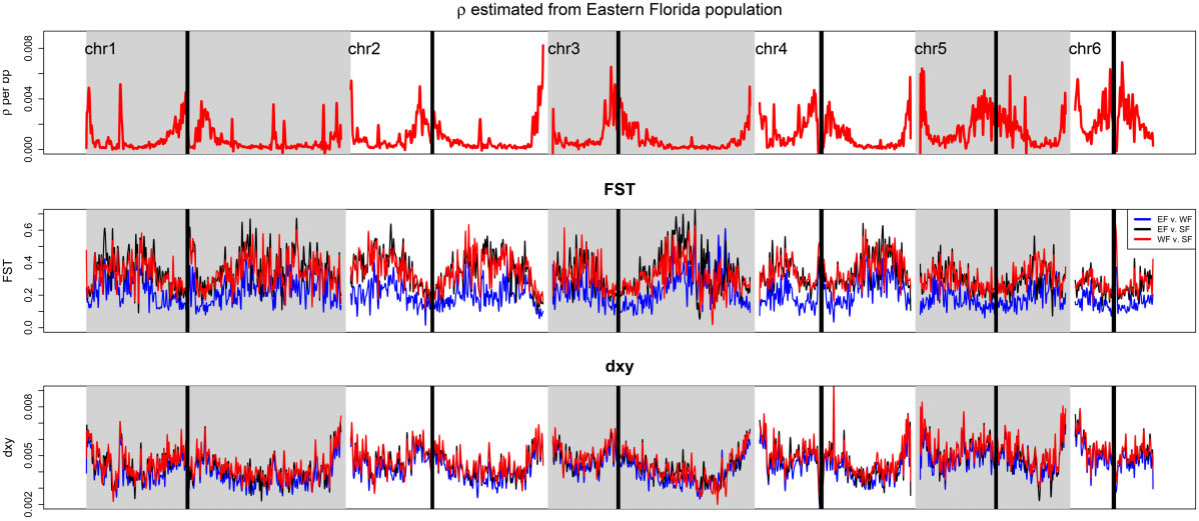
—Summary statistics for recombination and differentiation along chromosomes. *ρ *= 4×*N*_e_×*r*, with *r* the recombination rate per bp and per generation and *N*_e_ the effective population size for the EF cluster. *F*_ST_ and *d*_XY_ are relative and absolute measures of differentiation that are correlated with the amount of shared heterozygosity and coalescence time across populations, respectively. We present differentiation for the three genetic clusters having diverged for the longest time period. Statistics were averaged over nonoverlapping 5-kb windows and a smoothing line was fit to facilitate visual comparison. Repetitive centromeric regions that are masked from the green anole genome are highlighted by black rectangles.

Recombination rates estimated by LDHat in the EF cluster were highly heterogeneous along chromosomes, with stronger recombination rates at the tips and toward centromeres, though they dropped at the immediate vicinity of the latter (fig. 4). This pattern was supported by the Rozas’s *ZZ* statistic, suggesting stronger LD in the middle of chromosomes arms (supplementary fig. 6, Supplementary Material online). We observed higher relative differentiation (measured by *F*_ST_) in regions of low recombination (Spearman’s rank correlation test, all *P* values <2.2×10^−16^; fig. 4 and supplementary fig. 7, Supplementary Material online). The correlation was however opposite for measures of absolute differentiation (*d*_XY_), a statistics directly related to diversity and to the average age of alleles across populations (Cruickshank and Hahn 2014). These correlations are consistent with selection reducing heterozygosity in regions of low recombination, and further support the ∂a∂i models of heterogeneous effective population sizes along the genome. These measures of differentiation were strongly correlated when examining all three pairwise comparisons within Florida (fig. 4 and supplementary fig. 8, Supplementary Material online). There was also a positive correlation between recombination rate and the average frequency of derived alleles (DAF) in the EF cluster (Spearman’s rank correlation test, *ρ *= 0.11, *P* value < 0.001), although we did observe an increase in the average DAF for very low recombination rates that may be due to linked positive selection (supplementary fig. 9, Supplementary Material online). We did not observe any significant shift toward an excess of derived variants in regions of high divergence and low diversity (supplementary fig. 10, Supplementary Material online).

Given the possible effects of linked selection on diversity, we performed a SMC++ analysis masking 100-kb regions in the top 33% or low 33% *ρ/θ*_π_. We did not notice any strong deviation in the relative timing of divergence. The estimated effective population sizes remained in the same range of 1,000,000–5,000,000 individuals for each population in the last 3 Myr when compared with the unmasked analysis. Events more recent than ∼500 kya could not be reliably inferred due to masking. The strongest changes were observed for splitting times within Florida, for which estimates were ∼500,000 years younger with masking, with divergence between South Florida and other clades starting ∼2.3 Ma instead of 3.0 Ma.

## Discussion

In this study, we investigated the processes responsible for genomic differentiation and variation in the green anole. We showed that a complex history of recent expansion and SCs associated with linked selection shaped the genomic landscape of differentiation and diversity. Our results provide an important assessment of the forces acting on the green anole genome.

### A Dynamic Demographic History Has Shaped the Genomic Landscape of Differentiation

Green anole populations are strongly structured and it was hypothesized that successive splits and SC occurred in Florida during the Pleistocene (Tollis and Boissinot 2014; Manthey et al. 2016). Fluctuations in sea level may have generated temporary islands on which isolated populations could have diverged. At last, reconnection of Florida to the mainland would have provided the opportunity for expansion northward (Soltis et al. 2006). Our results support this claim in three ways. First, splitting times estimated by SMC++ and ∂a∂i suggest a series of splits in Florida between 3 and 2 Ma, a time range during which successions of glacial and interglacial periods may have led to several vicariance events (Lane 1994; Petuch 2004). Second, the models receiving the highest support in ∂a∂i were the ones allowing for several events of isolation followed by SC in Florida. Third, we found clear signatures of population expansion in GA and CA at the beginning of the Late Pleistocene, a time when lowering sea levels would have facilitated colonization (Lane 1994; Petuch 2004). We acknowledge that the exact timing of these events depends on the mutation rate used, which was previously established based on rates of divergence for three intronic nuclear markers compared with a mitochondrial one (Tollis and Boissinot 2014). The mutation rate used here is in the lower range of what is expected for nuclear markers compared with the mitochondrial rate of 1.3×10^−8^ per year commonly used in lizards (Macey et al. 1999), being ∼60 times lower while the average for squamates is ∼26 (Allio et al. 2017). Despite an old history of divergence, our ∂a∂i analysis found clear evidence for gene flow between taxa having diverged in the last 2 Myr. We note for example that ∂a∂i models with the highest likelihoods for the Gulf Atlantic-Eastern Florida comparison included heterogeneous migration rates along the genome, and suggested barriers to gene flow limiting introgression from Florida. This could reflect local adaptation through reduced effective migration rates at loci under selection in northern latitudes (but see Bierne et al. 2011).

While linked selection seems to play a major role in populations from Florida (see below), our results do not preclude the existence of heterogeneous gene flow along the genome, since we could not properly test the likelihood of models incorporating both of these aspects at once. Instead, they highlight the important role of linked selection in producing heterogeneous landscapes of differentiation (Cruickshank and Hahn 2014), even in a context of SC where genomic islands resisting gene flow may be expected. Recent years have seen a growing interest for the so-called “genomic islands of speciation,” regions that harbor higher differentiation than the genomic background (Feder and Nosil 2010; Ellegren et al. 2012; Nadeau et al. 2012; Wolf and Ellegren 2017). Several studies have since successfully highlighted the important role of heterogeneous migration and selection in shaping diversity in several organisms, such as mussels (Roux et al. 2014), sea bass (Tine et al. 2014), or poplars (Wang et al. 2016; Christe et al. 2017). This area of research has however been neglected so far in squamates, preventing any comparison of their genome dynamics at microevolutionary scales with other vertebrates. Our results call for more studies, for example using transects encompassing contact zones (Barton and Hewitt 1985), to assess how alleles diffuse across genetic clusters and better assess how heterogeneous gene flow may interact with recombination and linked selection to shape differentiation landscapes, which remains a challenging question (Nachman and Payseur 2012).

### Unequal Diversity between the X and Autosomal Chromosomes

We detected a significant deviation from a balanced ESR in the two populations that recently expanded and colonized North America, with strongly reduced nucleotide diversity on the X chromosome in the Gulf Atlantic population when compared with autosomal diversity. The method we used takes into account the expected difference in effective population sizes between autosomes and sex chromosomes. This suggests that the number of females that contributed to the present diversity on the X chromosome may have been extremely reduced compared with the number of males. Since this signature was found only in expanding populations, a possible explanation would be that the colonization of suboptimal habitats (compared with the center of origin in Florida) favored male-biased dispersal. The limited number of available females in the newly colonized regions would have therefore led to a biased sex-ratio in the founding populations and smaller effective population sizes on the X chromosome compared with unbiased expectations.

In *Anolis roquet*, male-biased dispersal is associated with competition, since males disperse more when density increases and competition for females is stronger (Johansson et al. 2008). In *Anolis sagrei*, smaller males tend to disperse more while females are more likely to stay in high quality territories, independently of female density (Calsbeek 2009). The green anole is a polygynous species, with sexual dimorphism and high levels of competition between males (Jenssen et al. 2000). It is therefore likely that competition within sexes may lead to unequal contribution of males and females to the gene pool.

Another nonexclusive possibility lies in the action of positive selection on the X chromosome in northern populations. The X chromosome is extremely small compared with autosomes in green anoles, probably not exceeding 20 Mb (Rupp et al. 2017). This means that even a few recent selective sweeps would have widespread effects on the entire chromosome, reducing diversity and the effective population size. Since the method implemented in KIMTREE compares estimates of effective population sizes between autosomes and X chromosome, this would result in an artificially biased sex-ratio. Sexual or natural selection may be responsible for this pattern, and our finding calls for further comparisons of sex-biased dispersal and behavior between populations of the green anole.

### Selection and Recombination Shape Nucleotide Composition and Diversity at Linked Sites

We observed strong heterogeneity in recombination rates along the green anole genome. Our results show that this heterogenous recombination landscape plays an important role in shaping genetic diversity in anoles. Both purifying and positive selections are expected to reduce diversity and increase genetic differentiation at linked sites (Cruickshank and Hahn 2014). We found evidence for an effect of linked selection in shaping differentiation between genetic clusters in Florida. These clusters have had relatively stable effective population sizes over the last 2 Myr, which should limit the stochasticity induced by drift in explaining heterogeneous patterns of differentiation along the genome. In that case, signatures of linked selection such as high differentiation and low diversity should be easier to detect in regions of low recombination. Indeed, regions of high diversity that are characterized by high *d*_XY_ displayed higher recombination rates and lower *F*_ST_ in the green anole, while regions with high *F*_ST_ were found in regions of low recombination and diversity. More specifically, a prominent role for background selection in shaping the differentiation landscape is supported by the correlations we observed between diversity, differentiation, and recombination. We acknowledge that positive selection may lead to similar signatures of high differentiation and low diversity in regions of low recombination due to the effects of selection at linked sites (Josephs and Wright 2016), and we did observe an increase in the average frequency of derived alleles in regions of very low recombination (supplementary fig. 9, Supplementary Material online). However, regions of both high divergence and low diversity did not seem to harbor any excess of derived alleles (supplementary fig. 10, Supplementary Material online) which suggests that positive selection is not the main driver of the differentiation landscape within Florida. Moreover, the agreement between landscapes of differentiation for all pairwise comparisons (fig. 4) suggests a reduction in diversity in the ancestral population rather than population-specific events of selection, consistent with background selection, or positive selection in the lineage ancestral to all green anoles.

Since green anoles constitute an important model species to understand the mechanisms of adaptation, adopting a cautious (i.e., nonadaptationist) interpretation of divergence landscapes is primordial (Burri 2017). Disentangling recent positive selection from the confounding effects of demography and background selection is especially challenging, even in species for which extensive genomic and functional studies have been performed, as in humans. Genome scans for positive selection have often failed to identify common outliers (Pavlidis et al. 2012), and require to carefully consider the demographic history of populations (Li et al. 2012; Pavlidis et al. 2012; Elyashiv et al. 2016; Schrider and Kern 2016; Sheehan and Song 2016; Bourgeois et al. 2018). The green anole is an important system to understand local adaptation in reptiles (Campbell-Staton et al. 2017) and the incorporation of our findings in future studies will be useful to properly test for signals of local adaptation. This will be done by taking into account the possible biases induced by demography and the impact of selection at linked sites. Further studies of positive selection will require more detailed analyses, building on the results we show in the present study.

## Supplementary Material

Supplementary data are available at *Genome Biology and Evolution* online.

## Supplementary Material

evz110_Supplementary_Data

## Notes

**Data deposition**: This project has been deposited to the Sequencing Read Archive under the Bioproject designation PRJNA533001.

## References

[evz110-B1] Alföldi J, et al 2011 The genome of the green anole lizard and a comparative analysis with birds and mammals. Nature 477(7366):587–591. 21881562 10.1038/nature10390 PMC3184186

[evz110-B2] Allio R, Donega S, Galtier N, Nabholz B. 2017 Large variation in the ratio of mitochondrial to nuclear mutation rate across animals: implications for genetic diversity and the use of mitochondrial DNA as a molecular marker. Mol Biol Evol. 34(11):2762–2772. 28981721 10.1093/molbev/msx197

[evz110-B3] Auton A, et al 2012 A fine-scale chimpanzee genetic map from population sequencing. Science 336(6078):193–198. 22422862 10.1126/science.1216872 PMC3532813

[evz110-B4] Barrett RDH, Rogers SM, Schluter D. 2008 Natural selection on a major armor gene in threespine stickleback. Science 322(5899):255–257. 18755942 10.1126/science.1159978

[evz110-B5] Barton NH, Hewitt GM. 1985 Analysis of hybrid zones. Annu Rev Ecol Syst. 16(1):113–148.

[evz110-B6] Begun DJ, Aquadro CF. 1992 Levels of naturally occurring DNA polymorphism correlate with recombination rates in *D. melanogaster*. Nature 356(6369):519–520. 1560824 10.1038/356519a0

[evz110-B7] Bierne N, Welch J, Loire E, Bonhomme F, David P. 2011 The coupling hypothesis: why genome scans may fail to map local adaptation genes. Mol Ecol. 20(10):2044–2072. 21476991 10.1111/j.1365-294X.2011.05080.x

[evz110-B302] Bolger AM, Lohse M, Usadel B. 2014. Trimmomatic: a flexible trimmer for Illumina sequence data. Bioinformatics 30(15):2114–2120. 10.1093/bioinformatics/btu170 PMC4103590 24695404

[evz110-B8] Booker TR, Ness RW, Keightley PD. 2017 The recombination landscape in wild house mice inferred using population genomic data. Genetics 207(1):297–309. 28751421 10.1534/genetics.117.300063 PMC5586380

[evz110-B9] Bourgeois Y, et al 2018 Genome-wide scans of selection highlight the impact of biotic and abiotic constraints in natural populations of the model grass *Brachypodium distachyon*. Plant J. 96(2):438–451. 30044522 10.1111/tpj.14042

[evz110-B10] Burri R. 2017 Interpreting differentiation landscapes in the light of long-term linked selection. Evol Lett. 1(3):118–131.

[evz110-B11] Burri R, et al 2015 Linked selection and recombination rate variation drive the evolution of the genomic landscape of differentiation across the speciation continuum of *Ficedula* flycatchers. Genome Res. 25(11):1656–1665. 26355005 10.1101/gr.196485.115 PMC4617962

[evz110-B12] Cai JJ, Macpherson JM, Sella G, Petrov DA. 2009 Pervasive hitchhiking at coding and regulatory sites in humans. PloS Genet 5(1):e1000336. 10.1371/journal.pgen.1000336 PMC2613029 19148272

[evz110-B13] Calsbeek R. 2009 Sex-specific adult dispersal and its selective consequences in the brown anole, *Anolis sagrei*. J Anim Ecol. 78(3):617–624. 19302322 10.1111/j.1365-2656.2009.01527.x

[evz110-B14] Campbell-Staton SC, Bare A, Losos JB, Edwards SV, Cheviron ZA. 2018 Physiological and regulatory underpinnings of geographic variation in reptilian cold tolerance across a latitudinal cline. Mol Ecol. 27(9):2243–2255. 29633453 10.1111/mec.14580

[evz110-B15] Campbell-Staton SC, Edwards SV, Losos JB. 2016 Climate-mediated adaptation after mainland colonization of an ancestrally subtropical island lizard, *Anolis carolinensis*. J Evol Biol. 29(11):2168–2180. 27384884 10.1111/jeb.12935

[evz110-B16] Campbell-Staton SC, et al 2012 Out of Florida: mtDNA reveals patterns of migration and pleistocene range expansion of the green anole lizard (*Anolis carolinensis*). Ecol Evol. 2(9):2274–2284. 23139885 10.1002/ece3.324 PMC3488677

[evz110-B17] Campbell-Staton SC, et al 2017 Winter storms drive rapid phenotypic, regulatory, and genomic shifts in the green anole lizard. Science 357(6350):495–498. 28774927 10.1126/science.aam5512

[evz110-B18] Christe C, et al 2017 Adaptive evolution and segregating load contribute to the genomic landscape of divergence in two tree species connected by episodic gene flow. Mol Ecol. 26(1):59–76. 27447453 10.1111/mec.13765

[evz110-B19] Clemente F, Gautier M, Vitalis R. 2018 Inferring sex-specific demographic history from SNP data. PLoS Genet. 14:1–32. 10.1371/journal.pgen.1007191 PMC5809101 29385127

[evz110-B20] Coffman AJ, Hsieh PH, Gravel S, Gutenkunst RN. 2016 Computationally efficient composite likelihood statistics for demographic inference. Mol Biol Evol. 33(2):591–593. 26545922 10.1093/molbev/msv255 PMC5854098

[evz110-B21] Costantini M, Greif G, Alvarez-Valin F, Bernardi G. 2016 The *Anolis* lizard genome: an amniote genome without isochores? Genome Biol Evol. 8(4):1048–1055. 26992416 10.1093/gbe/evw056 PMC4860688

[evz110-B22] Cruickshank TE, Hahn MW. 2014 Reanalysis suggests that genomic islands of speciation are due to reduced diversity, not reduced gene flow. Mol Ecol. 23(13):3133–3157. 24845075 10.1111/mec.12796

[evz110-B23] Danecek P, et al 2011 The variant call format and VCFtools. Bioinformatics 27(15):2156–2158. 21653522 10.1093/bioinformatics/btr330 PMC3137218

[evz110-B24] Depristo MA, et al 2011 A framework for variation discovery and genotyping using next-generation DNA sequencing data. Nat Genet. 43(5):491–501. 21478889 10.1038/ng.806 PMC3083463

[evz110-B25] Ellegren H, et al 2012 The genomic landscape of species divergence in Ficedula flycatchers. Nature 491(7426):756–760. 23103876 10.1038/nature11584

[evz110-B26] Elyashiv E, et al 2016 A genomic map of the effects of linked selection in Drosophila. PLoS Genet. 12:1–24. 10.1371/journal.pgen.1006130 PMC4990265 27536991

[evz110-B27] Excoffier L, Dupanloup I, Huerta-Sanchez E, Sousa VC, Foll M. 2013 Robust demographic inference from genomic and SNP data. PloS Genet 9(10): e1003905. 10.1371/journal.pgen.1003905 PMC3812088 24204310

[evz110-B28] Feder JL, Nosil P. 2010 The efficacy of divergence hitchhiking in generating genomic islands during ecological speciation. Evolution 64(6):1729–1747. 20624183 10.1111/j.1558-5646.2010.00943.x

[evz110-B29] Figuet E, Ballenghien M, Romiguier J, Galtier N. 2015 Biased gene conversion and GC-content evolution in the coding sequences of reptiles and vertebrates. Genome Biol Evol. 7(1):240–250. 10.1093/gbe/evu277 PMC4316630 25527834

[evz110-B30] Fujita MK, Edwards SV, Ponting CP. 2011 The *Anolis* lizard genome: an amniote genome without isochores. Genome Biol Evol. 3:974–984. 21795750 10.1093/gbe/evr072 PMC3184785

[evz110-B31] Gautier M, Vitalis R. 2013 Inferring population histories using genome-wide allele frequency data. Mol Biol Evol. 30(3):654–668. 23155004 10.1093/molbev/mss257

[evz110-B32] Glor RE, Losos JB, Larson A. 2005 Out of Cuba: overwater dispersal and speciation among lizards in the *Anolis carolinensis* subgroup. Mol Ecol. 14(8):2419–2432. 15969724 10.1111/j.1365-294X.2005.02550.x

[evz110-B33] Gutenkunst RN, Hernandez RD, Williamson SH, Bustamante CD. 2009 Inferring the joint demographic history of multiple populations from multidimensional SNP frequency data. PloS Genet 5(10): e1000695. 10.1371/journal.pgen.1000695 PMC2760211 19851460

[evz110-B34] Han F, et al 2017 Gene flow, ancient polymorphism, and ecological adaptation shape the genomic landscape of divergence among Darwin’s finches. Genome Res. 27(6):1004–1015. 28442558 10.1101/gr.212522.116 PMC5453315

[evz110-B35] Huson DH, Bryant D. 2006 Application of phylogenetic networks in evolutionary studies. Mol Biol Evol. 23(2):254–267. 16221896 10.1093/molbev/msj030

[evz110-B36] Jensen JD, et al 2019 The importance of the Neutral Theory in 1968 and 50 years on: a response to Kern and Hahn 2018. Evolution 73(1):111–114. 30460993 10.1111/evo.13650 PMC6496948

[evz110-B37] Jenssen T, Orrell KS, Lovern MB, Ross S. 2000 Sexual dimorphisms in aggressive signal structure and use by a polygynous lizard, *Anolis carolinensis*. Copeia 2000(1):140–149.

[evz110-B38] Johansson H, Surget-Groba Y, Thorpe RS. 2008 Microsatellite data show evidence for male-biased dispersal in the Caribbean lizard *Anolis roquet*. Mol Ecol. 17(20):4425–4432. 18803592 10.1111/j.1365-294X.2008.03923.x

[evz110-B39] Jombart T, et al 2010 Discriminant analysis of principal components: a new method for the analysis of genetically structured populations. BMC Genet. 11(1):94. 20950446 10.1186/1471-2156-11-94 PMC2973851

[evz110-B40] Josephs EB, Wright SI. 2016 On the trail of linked selection. PLoS Genet. 12:1–5. 10.1371/journal.pgen.1006240 PMC4990175 27537331

[evz110-B41] Kawakami T, et al 2017 Whole-genome patterns of linkage disequilibrium across flycatcher populations clarify the causes and consequences of fine-scale recombination rate variation in birds. Mol Ecol. 26(16):4158–4172. 28597534 10.1111/mec.14197

[evz110-B42] Kern AD, Hahn MW. 2018 The neutral theory in light of natural selection. Mol Biol Evol. 35(6):1366–1371. 29722831 10.1093/molbev/msy092 PMC5967545

[evz110-B43] Kolbe JJ, et al 2017 An incipient invasion of brown anole lizards (*Anolis sagrei*) into their own native range in the Cayman Islands: a case of cryptic back-introduction. Biol Invasions. 19(7):1989–1998.

[evz110-B44] Lailvaux SP, Herrel A, Vanhooydonck B, Meyers JJ, Irschick DJ. 2004 Performance capacity, fighting tactics and the evolution of life-stage male morphs in the green anole lizard (*Anolis carolinensis*). Proc Biol Sci. 271(1556):2501–2508. 15590602 10.1098/rspb.2004.2891 PMC1691885

[evz110-B401] Lane, E. 1994. Florida's Geological History and Geological Resources. Special publication (Florida Geological Survey (1989)). Vol. 35. Published for the Florida Geological Survey; Tallahassee, FL.

[evz110-B45] Lapiedra O, Schoener TW, Leal M, Losos JB, Kolbe JJ. 2018 Predator-driven natural selection on risk-taking behavior in anole lizards. Science 360(6392):1017–1020. 29853685 10.1126/science.aap9289

[evz110-B46] Larson EL, White TA, Ross CL, Harrison RG. 2014 Gene flow and the maintenance of species boundaries. Mol Ecol. 23(7):1668–1678. 24795995 10.1111/mec.12601

[evz110-B47] Li H, Durbin R. 2011 Inference of human population history from individual whole-genome sequences. Nature 475(7357):493–496. 21753753 10.1038/nature10231 PMC3154645

[evz110-B48] Li H, et al 2009 The Sequence Alignment/Map format and SAMtools. Bioinformatics 25(16):2078–2079. 19505943 10.1093/bioinformatics/btp352 PMC2723002

[evz110-B49] Li J, et al 2012 Joint analysis of demography and selection in population genetics: where do we stand and where could we go? Mol Ecol. 21(1):28–44. 21999307 10.1111/j.1365-294X.2011.05308.x

[evz110-B50] Losos JB. 2009 Lizards in an evolutionary tree. Berkeley: University of California Press [Database]

[evz110-B51] Losos JB, Schoener TW, Spiller DA. 2004 Predator-induced behaviour shifts and natural selection in field-experimental lizard populations. Nature 432(7016):505–508. 15565155 10.1038/nature03039

[evz110-B52] Macey JR, et al 1999 Molecular phylogenetics, tRNA evolution, and historical biogeography in Anguid lizards and related taxonomic families. Mol Phylogenet Evol. 12(3):250–272. 10413621 10.1006/mpev.1999.0615

[evz110-B53] Malinsky M, et al 2015 Genomic islands of speciation separate cichlid ecomorphs in an East African crater lake. Science 350(6267):1493–1498. 26680190 10.1126/science.aac9927 PMC4700518

[evz110-B54] Manthey JD, Tollis M, Lemmon AR, Moriarty Lemmon E, Boissinot S. 2016 Diversification in wild populations of the model organism *Anolis carolinensis*: a genome-wide phylogeographic investigation. Ecol Evol. 6(22):8115–8125. 27891220 10.1002/ece3.2547 PMC5108263

[evz110-B55] McGee MD, Neches RY, Seehausen O. 2016 Evaluating genomic divergence and parallelism in replicate ecomorphs from young and old cichlid adaptive radiations. Mol Ecol. 25(1):260–268. 26558354 10.1111/mec.13463

[evz110-B56] McVean G, Awadalla P, Fearnhead P. 2002 A coalescent-based method for detecting and estimating recombination from gene sequences. Genetics 160(3):1231–1241. 11901136 10.1093/genetics/160.3.1231 PMC1462015

[evz110-B57] Mullen LM, Hoekstra HE. 2008 Natural selection along an environmental gradient: a classic cline in mouse pigmentation. Evolution 62(7):1555–1570. 18489719 10.1111/j.1558-5646.2008.00425.x

[evz110-B58] Nachman MW, Payseur BA. 2012 Recombination rate variation and speciation: theoretical predictions and empirical results from rabbits and mice. Philos Trans R Soc Lond B Biol Sci. 367(1587):409–421. 22201170 10.1098/rstb.2011.0249 PMC3233716

[evz110-B59] Nadeau NJ, et al 2012 Genomic islands of divergence in hybridizing *Heliconius* butterflies identified by large-scale targeted sequencing. Philos Trans R Soc Lond B Biol Sci. 367(1587):343–353. 22201164 10.1098/rstb.2011.0198 PMC3233711

[evz110-B60] Noor MAF, Bennett SM. 2009 Islands of speciation or mirages in the desert Examining the role of restricted recombination in maintaining species. Heredity 103(6):439–444. 19920849 10.1038/hdy.2009.151 PMC2809014

[evz110-B61] Pavlidis P, Jensen JD, Stephan W, Stamatakis A. 2012 A critical assessment of storytelling: gene ontology categories and the importance of validating genomic scans. Mol Biol Evol. 29(10):3237–3248. 22617950 10.1093/molbev/mss136

[evz110-B62] Payseur BA, Krenz JG, Nachman MW. 2004 Differential patterns of introgression across the X chromosome in a hybrid zone between two species of house mice. Evolution 58(9):2064–2078. 15521462 10.1111/j.0014-3820.2004.tb00490.x

[evz110-B301] Petuch, EJ. 2004. Cenozoic Seas : the View from Eastern North America. Boca Raton: CRC Press.

[evz110-B63] Pfeifer B, Wittelsburger U, Ramos-Onsins SE, Lercher MJ. 2014 PopGenome: an efficient Swiss army knife for population genomic analyses in R. Mol Biol Evol. 31(7):1929–1936. 24739305 10.1093/molbev/msu136 PMC4069620

[evz110-B64] Poelstra JW, et al 2014 The genomic landscape underlying phenotypic integrity in the face of gene flow in crows. Science 344(6190):1410–1414. 24948738 10.1126/science.1253226

[evz110-B65] Pouyet F, Aeschbacher S, Thiéry A, Excoffier L. 2018 Background selection and biased gene conversion affect more than 95% of the human genome and bias demographic inferences. Elife 7:1–21. 10.7554/eLife.36317 PMC6177262 30125248

[evz110-B66] Quinlan AR, Hall IM. 2010 BEDTools: a flexible suite of utilities for comparing genomic features. Bioinformatics 26(6):841–842. 20110278 10.1093/bioinformatics/btq033 PMC2832824

[evz110-B67] Roux C, et al 2014 Can we continue to neglect genomic variation in introgression rates when inferring the history of speciation? A case study in a Mytilus hybrid zone. J Evol Biol. 27(8):1662–1675. 24913446 10.1111/jeb.12425

[evz110-B68] Roux C, et al 2016 Shedding light on the grey zone of speciation along a continuum of genomic divergence. PLoS Biol 14(12):e2000234. 10.1371/journal.pbio.2000234 PMC5189939 28027292

[evz110-B69] Rozas J, Gullaud M, Blandin G, Aguade M. 2001 DNA variation at the *rp49* gene region of *Drosophila simulans*: evolutionary inferences from an unusual haplotype structure. Genetics 158(3):1147–1155. 11454763 10.1093/genetics/158.3.1147 PMC1461709

[evz110-B70] Ruggiero RP, Bourgeois Y, Boissinot S. 2017 LINE insertion polymorphisms are abundant but at low frequencies across populations of *Anolis carolinensis*. Front Genet. 8:1–14. 28450881 10.3389/fgene.2017.00044 PMC5389967

[evz110-B71] Rupp SM, et al 2017 Evolution of dosage compensation in *Anolis carolinensis*, a reptile with XX/XY chromosomal sex determination. Genome Biol Evol. 9(1):231–240. 28206607 10.1093/gbe/evw263 PMC5381669

[evz110-B72] Schrider DR, Kern AD. 2016 S/HIC: robust identification of soft and hard sweeps using machine learning. PLoS Genet. 12:1–31. 10.1371/journal.pgen.1005928 PMC4792382 26977894

[evz110-B73] Seehausen O, et al 2014 Genomics and the origin of species. Nat Rev Genet. 15(3):176–192. 24535286 10.1038/nrg3644

[evz110-B74] Sheehan S, Song YS. 2016 Deep learning for population genetic inference. PLoS Comput Biol. 12:1–28. 10.1371/journal.pcbi.1004845 PMC4809617 27018908

[evz110-B75] Soltis DE, Morris AB, McLachlan JS, Manos PS, Soltis PS. 2006 Comparative phylogeography of unglaciated eastern North America. Mol Ecol. 15(14):4261–4293. 17107465 10.1111/j.1365-294X.2006.03061.x

[evz110-B76] Sousa VC, Carneiro M, Ferrand N, Hey J. 2013 Identifying loci under selection against gene flow in isolation-with-migration models. Genetics 194(1):211–233. 23457232 10.1534/genetics.113.149211 PMC3632470

[evz110-B77] Stamatakis A. 2014 RAxML version 8: a tool for phylogenetic analysis and post-analysis of large phylogenies. Bioinformatics 30(9):1312–1313. 24451623 10.1093/bioinformatics/btu033 PMC3998144

[evz110-B78] Tajima F. 1989 Statistical method for testing the neutral mutation hypothesis by DNA polymorphism. Genetics 123(3):585–595. 2513255 10.1093/genetics/123.3.585 PMC1203831

[evz110-B79] Teeter KC, et al 2008 Genome-wide patterns of gene flow across a house mouse hybrid zone. Genome Res. 18(1):67–76., 18025268 10.1101/gr.6757907 PMC2134771

[evz110-B80] Terhorst J, Kamm JA, Song YS. 2017 Robust and scalable inference of population history from hundreds of unphased whole genomes. Nat Genet. 49(2):303–309. 28024154 10.1038/ng.3748 PMC5470542

[evz110-B81] Tine M, et al 2014 European sea bass genome and its variation provide insights into adaptation to euryhalinity and speciation. Nat Commun. 5:5770. 25534655 10.1038/ncomms6770 PMC4284805

[evz110-B82] Tollis M, Ausubel G, Ghimire D, Boissinot S. 2012 Multi-locus phylogeographic and population genetic analysis of *Anolis carolinensis*: historical demography of a genomic model species. PLoS One 7:1–14. 10.1371/journal.pone.0038474 PMC3369884 22685573

[evz110-B83] Tollis M, Boissinot S. 2011 The transposable element profile of the *Anolis* genome: how a lizard can provide insights into the evolution of vertebrate genome size and structure. Mob Genet Elements. 1(2):107–111. 22016857 10.4161/mge.1.2.17733 PMC3190321

[evz110-B84] Tollis M, Boissinot S. 2013 Lizards and LINEs: selection and demography affect the fate of L1 retrotransposons in the genome of the green anole (*Anolis carolinensis*). Genome Biol Evol. 5(9):1754–1768. 24013105 10.1093/gbe/evt133 PMC3787681

[evz110-B85] Tollis M, Boissinot S. 2014 Genetic variation in the green anole lizard (*Anolis carolinensis*) reveals island refugia and a fragmented florida during the quaternary. Genetica 1:59–72. 10.1007/s10709-013-9754-1 PMC4778398 24379168

[evz110-B86] Torres R, Stetter MG, Hernandez RD, Ross-Ibarra J. 2019. The temporal dynamics of background selection in non-equilibrium populations. bioarXiv. doi: https://doi.org/10.1101/618389 10.1534/genetics.119.302892 PMC7153942 32071195

[evz110-B87] Van Der Auwera GA, et al 2014 From FastQ data to high confidence variant calls: the Genome Analysis Toolkit best practices pipeline. Curr Protoc Bioinforma. 11:11.10.1–11.10.33. 10.1002/0471250953.bi1110s43 PMC4243306 25431634

[evz110-B88] Wade J. 2012 Sculpting reproductive circuits: relationships among hormones, morphology and behavior in anole lizards. Gen Comp Endocrinol. 176(3):456–460. 22202602 10.1016/j.ygcen.2011.12.011

[evz110-B89] Wang J, Street NR, Scofield DG, Ingvarsson PK. 2016 Variation in linked selection and recombination drive genomic divergence during allopatric speciation of European and American aspens. Mol Biol Evol. 33(7):1754–1767. 26983554 10.1093/molbev/msw051 PMC4915356

[evz110-B90] Wolf JBW, Ellegren H. 2017 Making sense of genomic islands of differentiation in light of speciation. Nat Rev Genet. 18(2):87–100. 27840429 10.1038/nrg.2016.133

