# Supplementary material for: Recent Secondary Contacts, Linked Selection, and Variable Recombination Rates Shape Genomic Diversity in the Model Species Anolis carolinensis
Source: Genome Biol Evol. 2019 May 27;11(7):2009–22. doi: 10.1093/gbe/evz110 (PMC6681179; doi:10.1093/gbe/evz110)
Supplement: evz110_Supplementary_Data [file evz110_supplementary_data.zip › Supporting_Info_Legends.docx]

Supplementary Table

Table S1. Samples origin, sequencing depth and quality statistics.

Supplementary Figures

Figure S1: Plot of average depth of coverage for sex-linked markers v. autosomal markers in all 27 green anoles used in this study. Males should fall on the line y=2*x due to the representation bias expected in XY individuals. Females are XX and should fall on the line y=x.

Figure S2: LD decay over 100kb in Gulf Atlantic and NE Florida populations.

Figure S3: Bayesian Information Criterion (BIC) for each value of the number of cluster K inferred by DAPC. The K with the lowest BIC has the highest likelihood.

Figure S4: Boxplots of nucleotide diversity across non-overlapping 5kb windows at autosomes and sex-linked scaffolds for three EF females and three Gulf Atlantic females. The analysis was restricted to females to account for haplodiploidy at sex-linked scaffolds. The dotted lines delimit autosomes from sex-linked scaffolds.

Figure S5. Likelihoods obtained for the 34 ∂a∂i models in the EF v. GA and EF v. WF comparisons. Higher likelihoods suggest better support for a given model. Complexity was added to the models described in Figure 3A by including various combinations of population expansion (prefix ‘ex’), heterogeneous asymmetric migration rates (suffix ‘2M2P’) and heterogeneous effective population size (suffix ‘2N’) among loci.

Figure S6: Comparison between.LDHat estimates for Eastern Florida of *ρ*= 4**N_e_***r* and Rozas’s *ZZ*, which is a measure of linkage disequilibrium positively correlated to intragenic recombination.

Figure S7: Correlations between *ρ/ θ_π_*, *F_ST_*_,_ and *d_XY_* at the genome scale. Statistics were estimated over non-overlapping 100kb windows. Spearman’s ρ coefficients are indicated on the graphs.

Figure S8: Comparison of pairwise *F_ST_* and *d_XY_* for all three pairwise comparisons of clusters found in Florida.

Figure S9: Average derived allele frequency for 100kb windows in five increasing quantiles of recombination. Recombination was measured as the ratio *ρ/ θ_π._*

Figure S10: Distribution of the average derived allele frequency in NE Florida in 100kb windows. Outlier windows are defined as belonging to the top 20% *F_ST_* and bottom 20% *d_XY_* computed between NE Florida and South Florida or between NE Florida and NW Florida.
