# Supplementary material for: Recent Secondary Contacts, Linked Selection, and Variable Recombination Rates Shape Genomic Diversity in the Model Species Anolis carolinensis
Source: Genome Biol Evol. 2019 May 27;11(7):2009–22. doi: 10.1093/gbe/evz110 (PMC6681179; doi:10.1093/gbe/evz110)
Supplement: evz110_Supplementary_Data [file evz110_supplementary_data.zip › Sup_Figure_7.pdf]

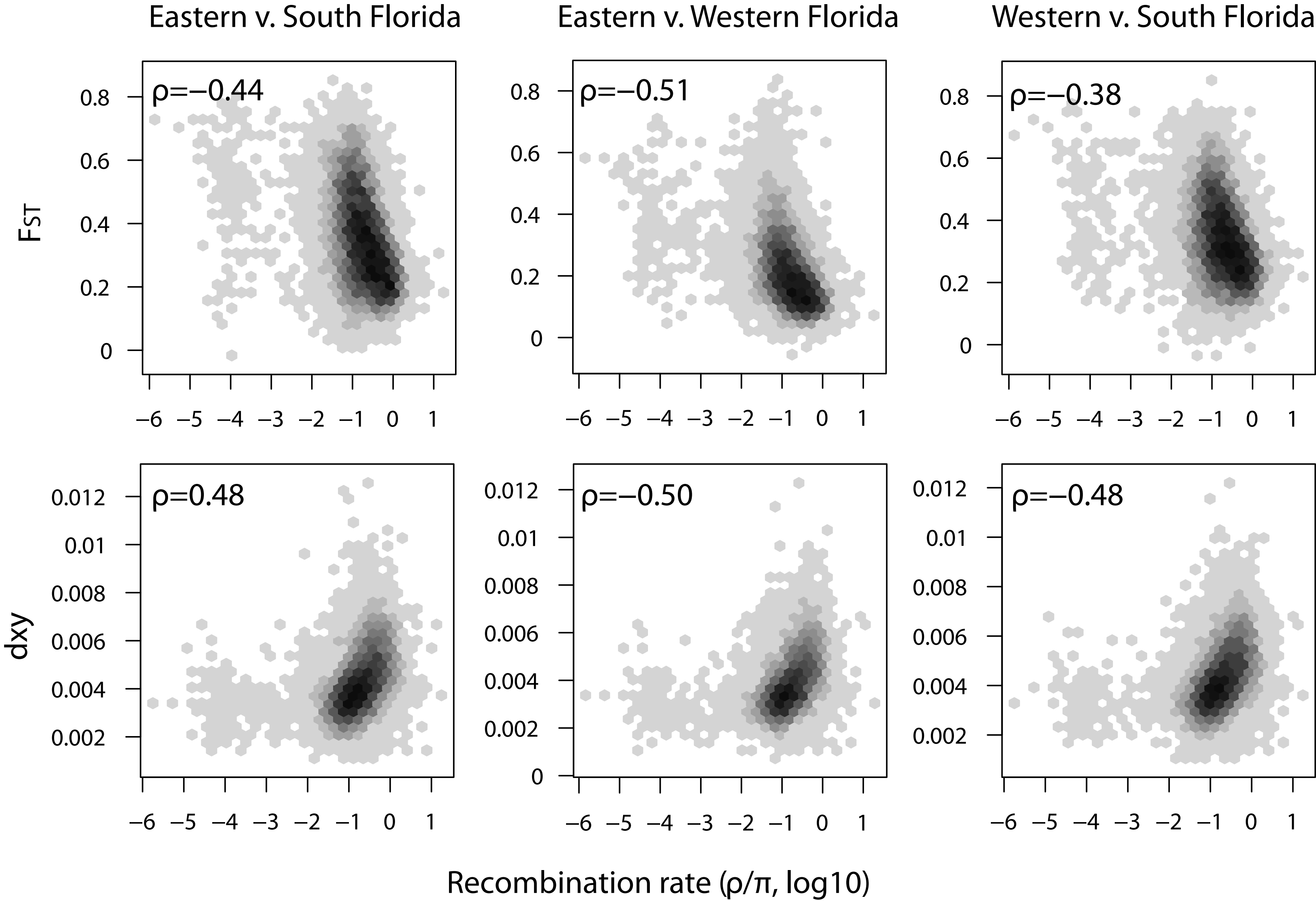
Eastern v. South Florida
Eastern v. Western Florida
Western v. South Florida
FST
dxy
ρ=−0.44
ρ=−0.51
ρ=−0.38
ρ=0.48
ρ=−0.50
ρ=−0.48
0.8
0.6
0.4
0.2
0
0.012
0.01
0.008
0.006
0.004
0.002
−6 −5 −4 −3 −2 −1 0 1
Recombination rate (ρ/π, log10)
