# Supplementary figures and images for: Recent Secondary Contacts, Linked Selection, and Variable Recombination Rates Shape Genomic Diversity in the Model Species Anolis carolinensis
Source: Genome Biol Evol. 2019 May 27;11(7):2009–22. doi: 10.1093/gbe/evz110 (PMC6681179; doi:10.1093/gbe/evz110)

# Average DAF in East Florida

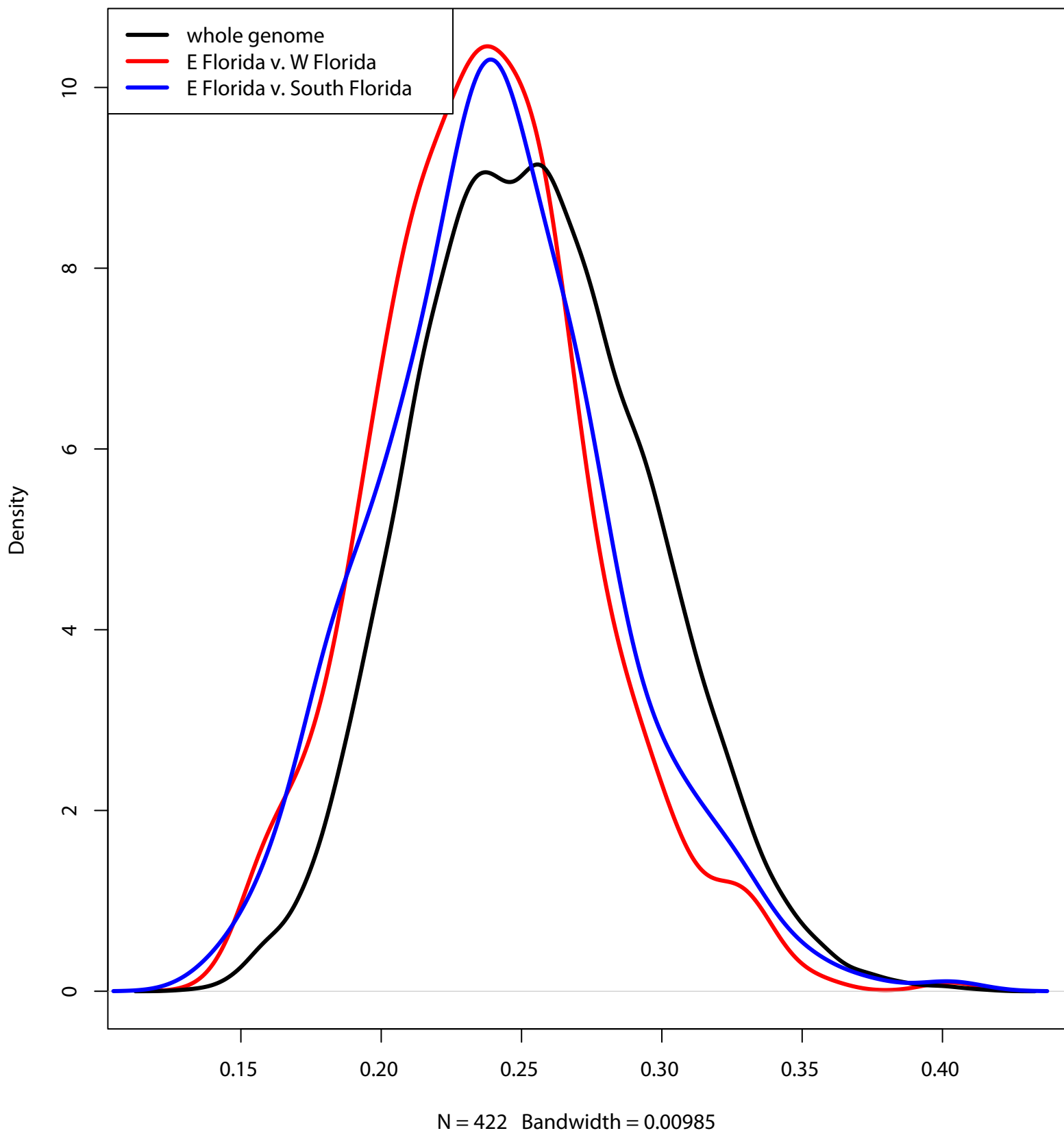

Supplement: evz110_Supplementary_Data [file evz110_supplementary_data.zip › Sup_Figure_10.pdf]

Eastern v. Western Florida

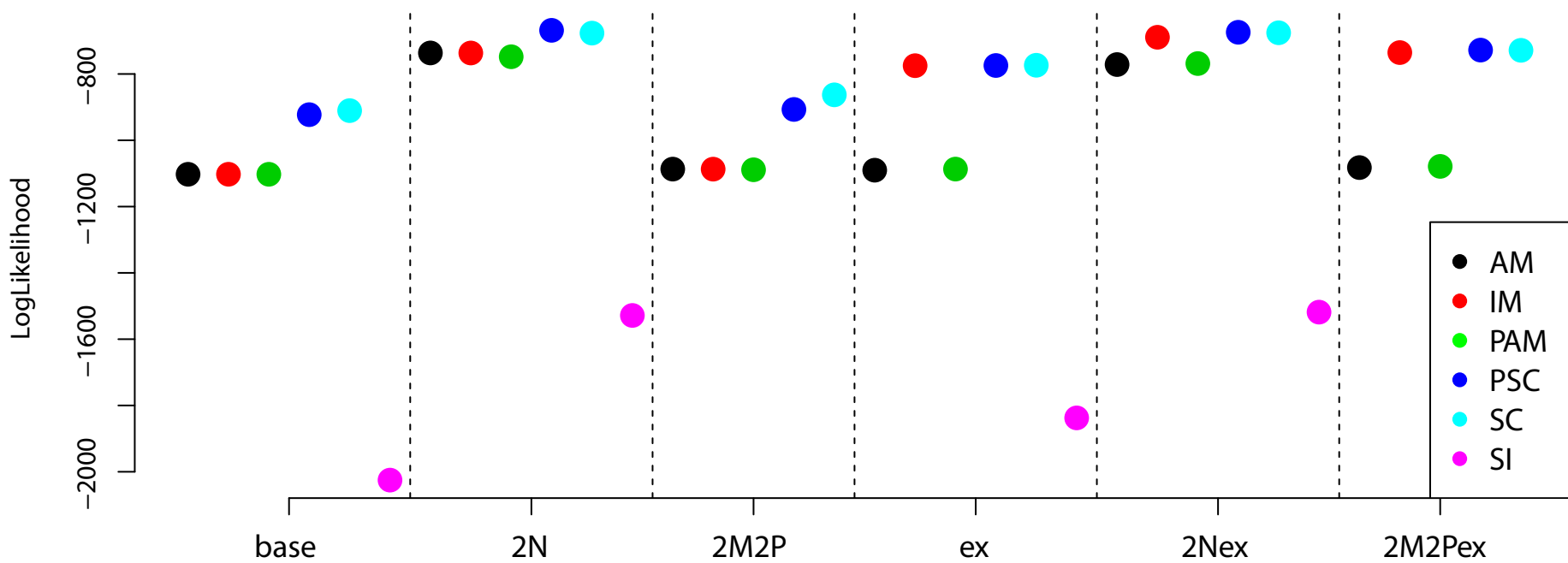

Eastern Florida v. Gulf Atlantic

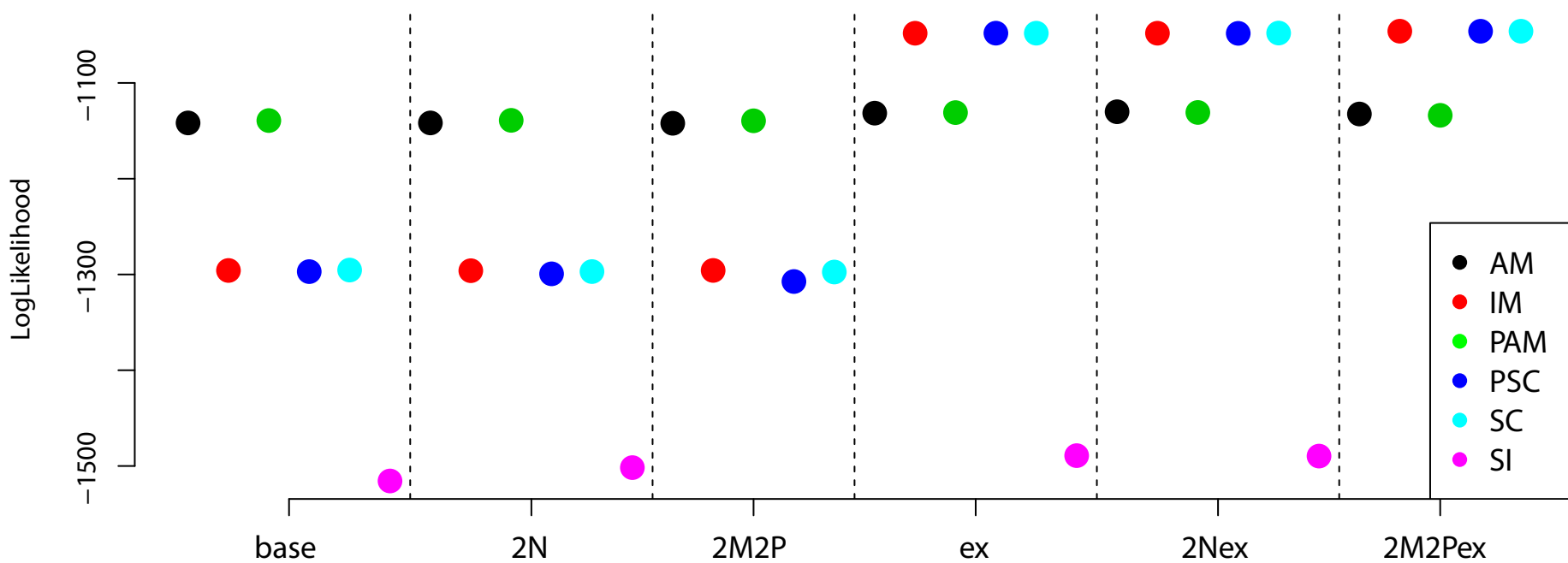

Supplement: evz110_Supplementary_Data [file evz110_supplementary_data.zip › Sup_Figure_5.pdf]

$\rho$  estimated from Eastern Florida population

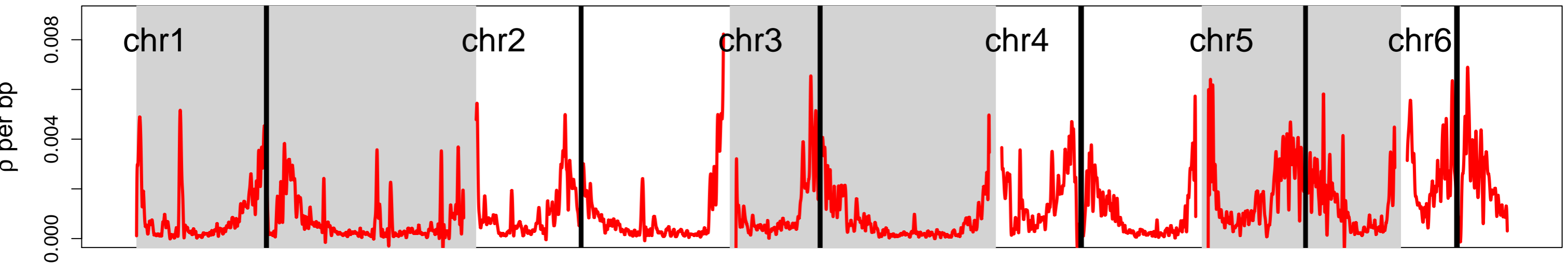

**Rozas's ZZ recombination statistics**

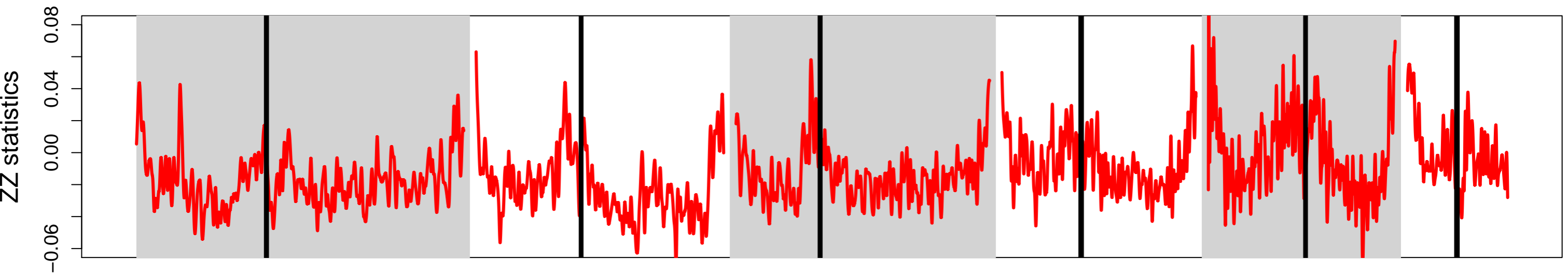

Supplement: evz110_Supplementary_Data [file evz110_supplementary_data.zip › Sup_Figure_6.pdf]

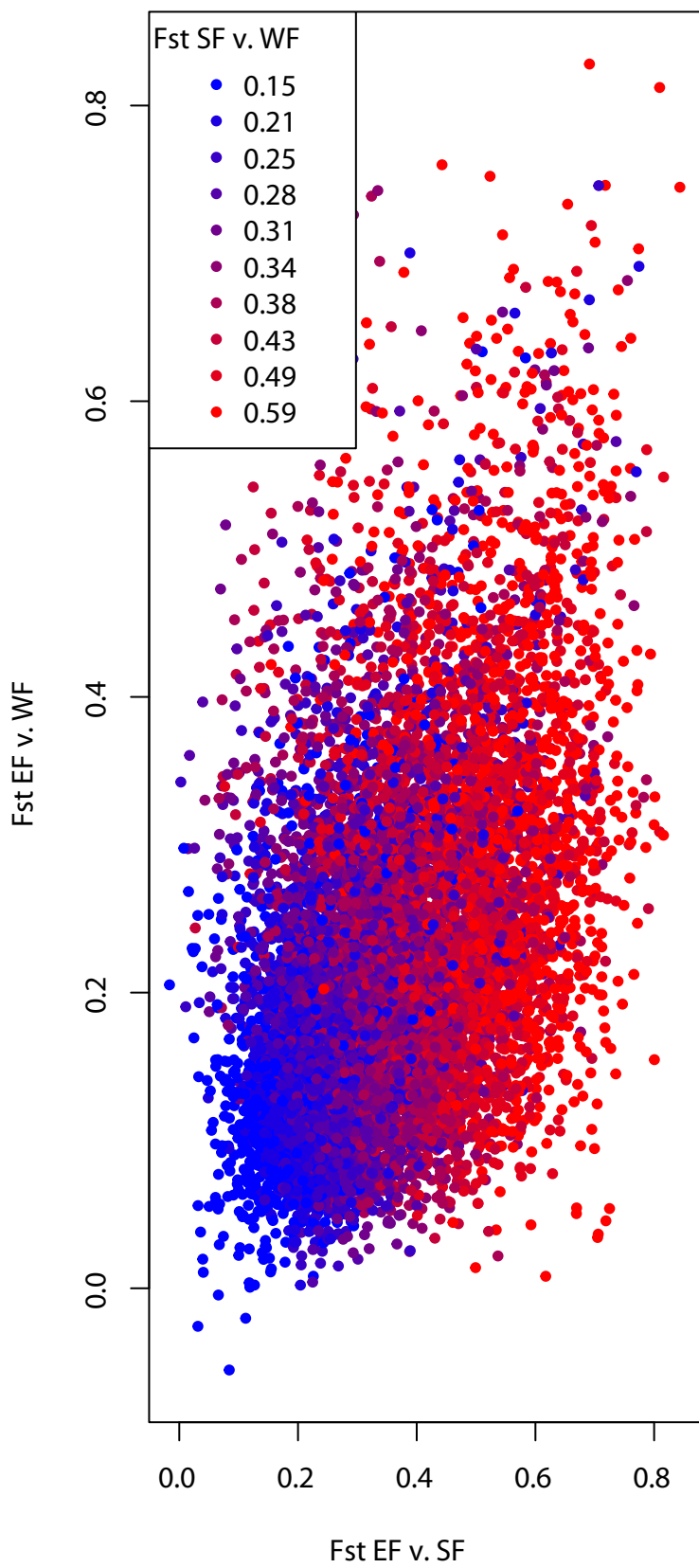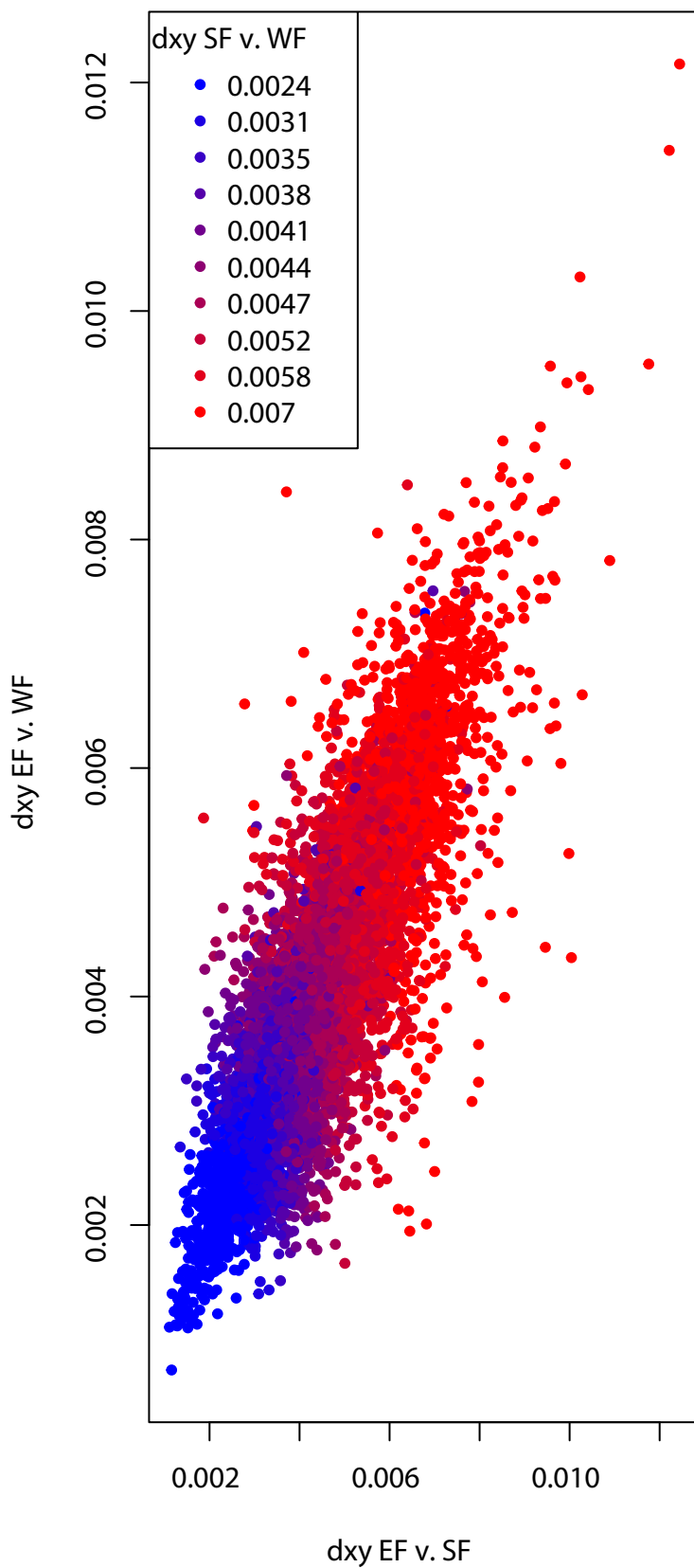

Supplement: evz110_Supplementary_Data [file evz110_supplementary_data.zip › Sup_figure_8.pdf]

Quantile #

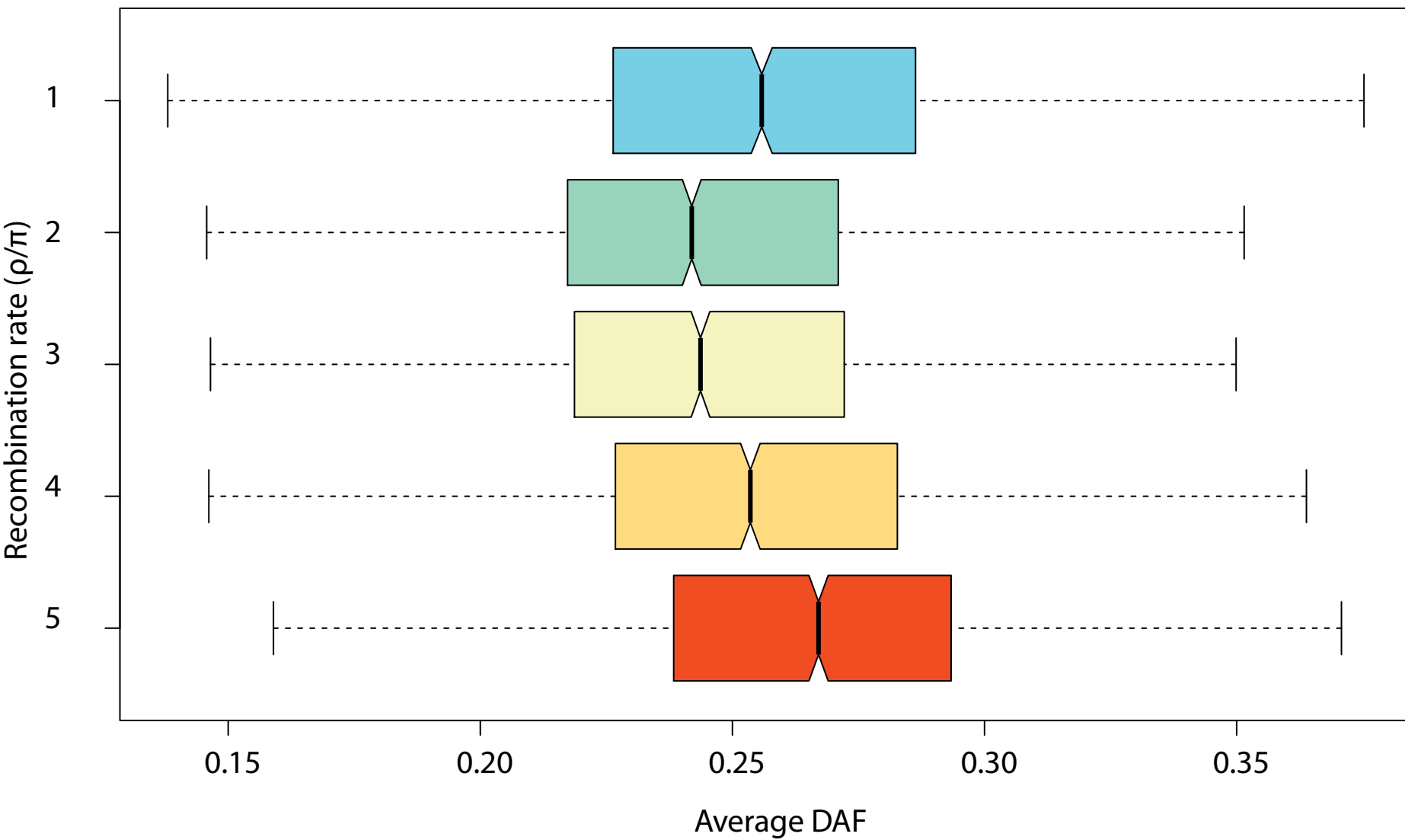

Supplement: evz110_Supplementary_Data [file evz110_supplementary_data.zip › Sup_figure_9.pdf]

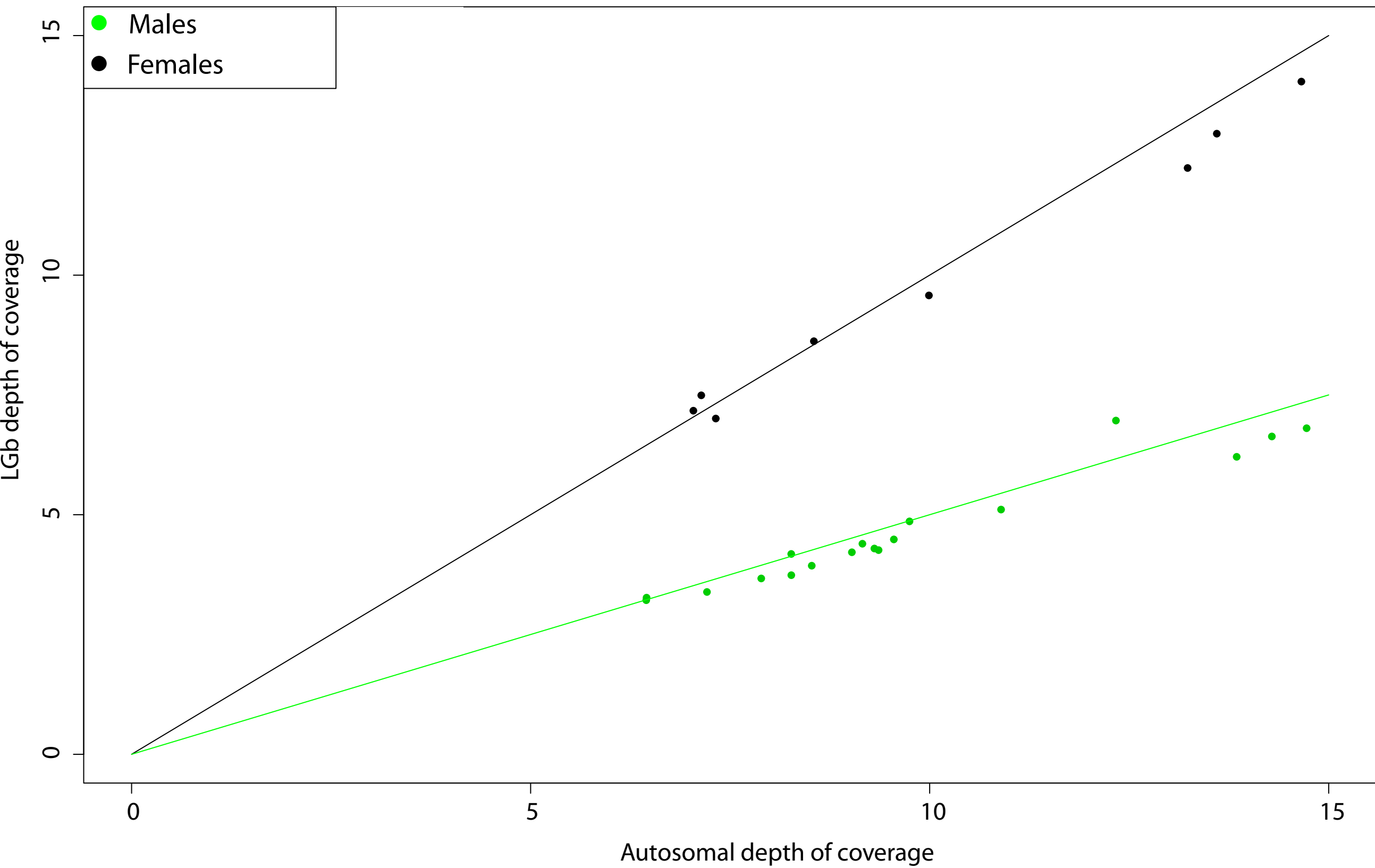

Supplement: evz110_Supplementary_Data [file evz110_supplementary_data.zip › Sup_Fig_1.pdf]

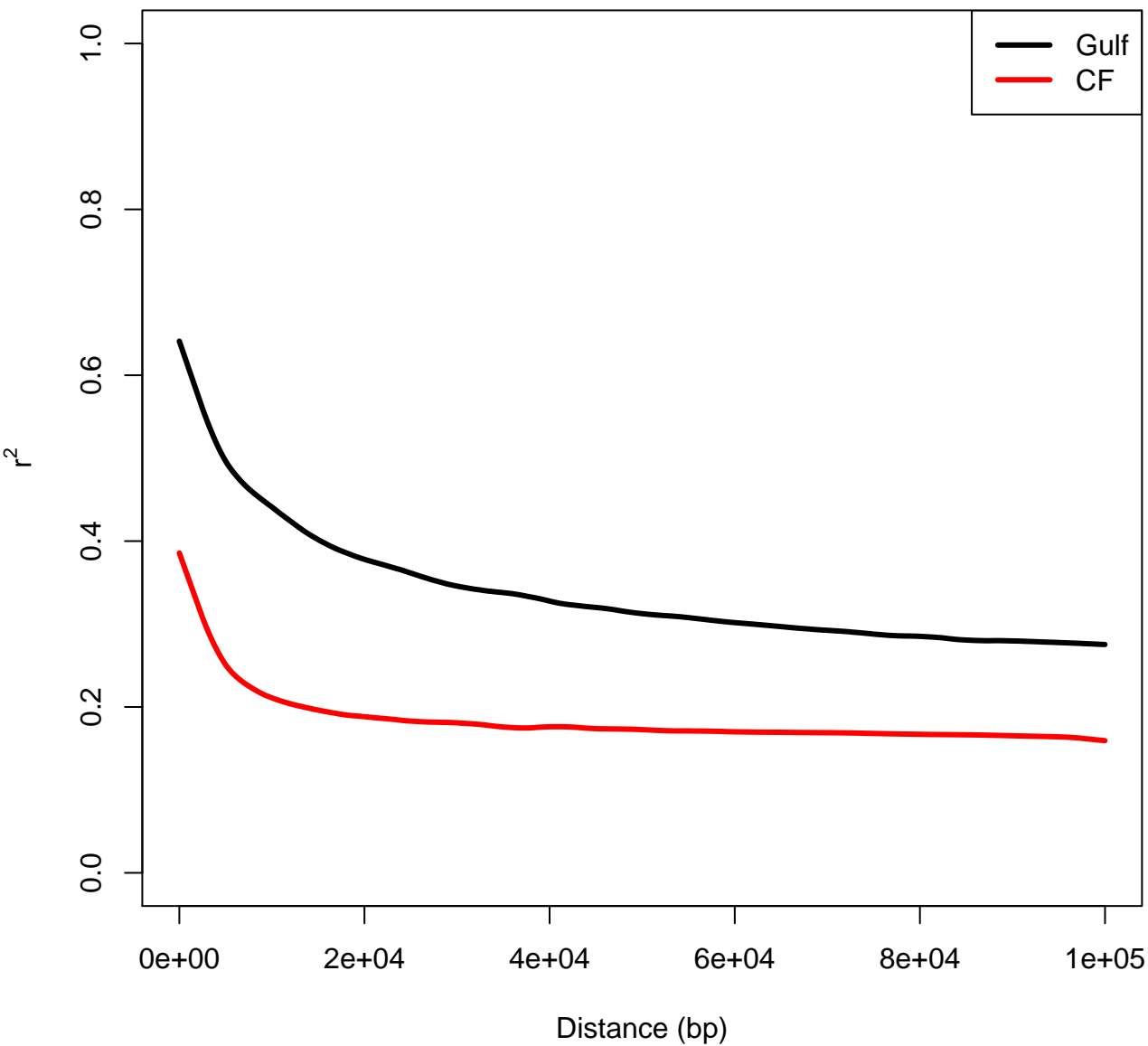

Supplement: evz110_Supplementary_Data [file evz110_supplementary_data.zip › Sup_Fig_2.pdf]

**Value of BIC  
versus number of clusters**

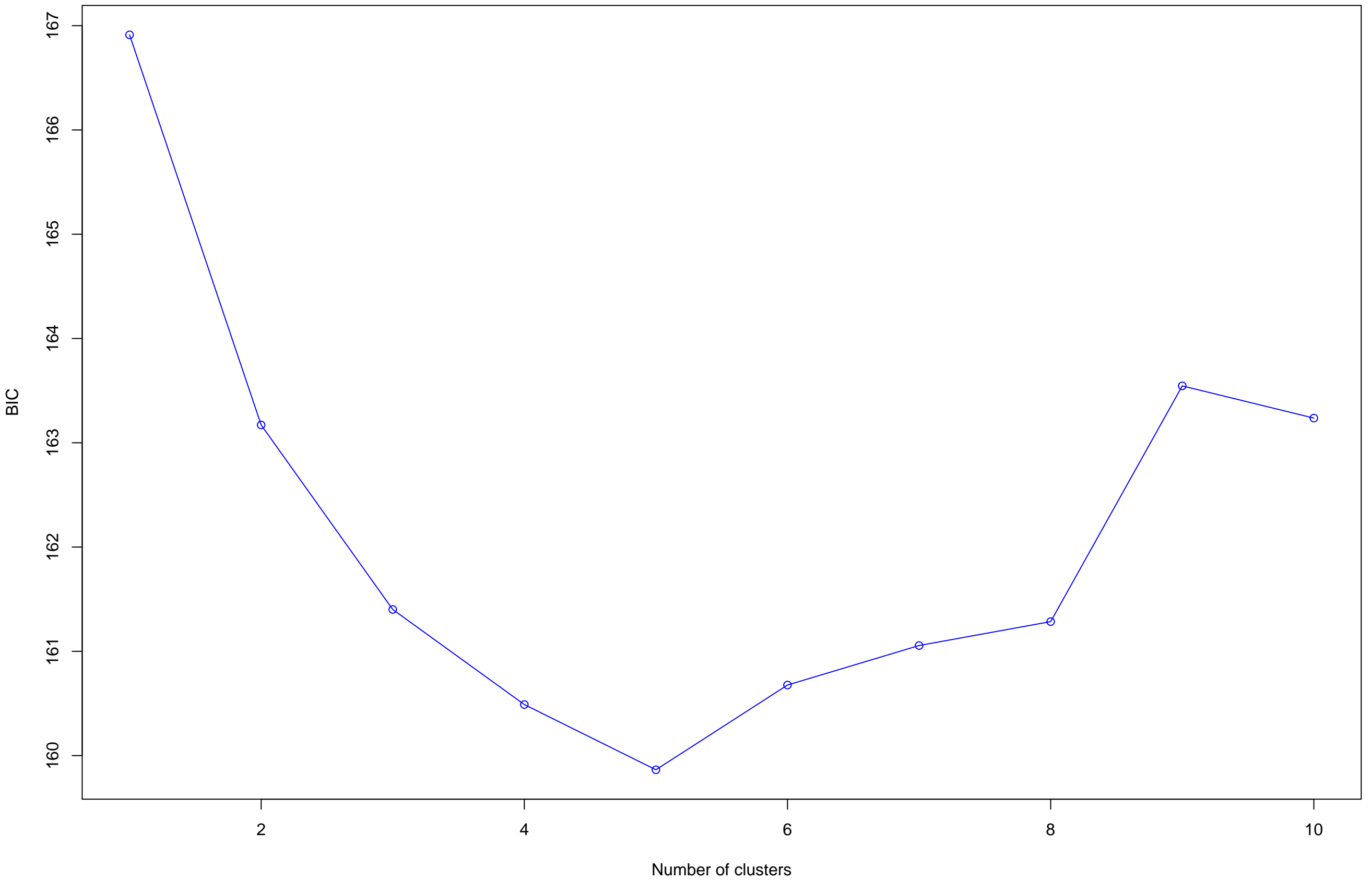

Supplement: evz110_Supplementary_Data [file evz110_supplementary_data.zip › Sup_Fig_3.pdf]

Eastern Florida

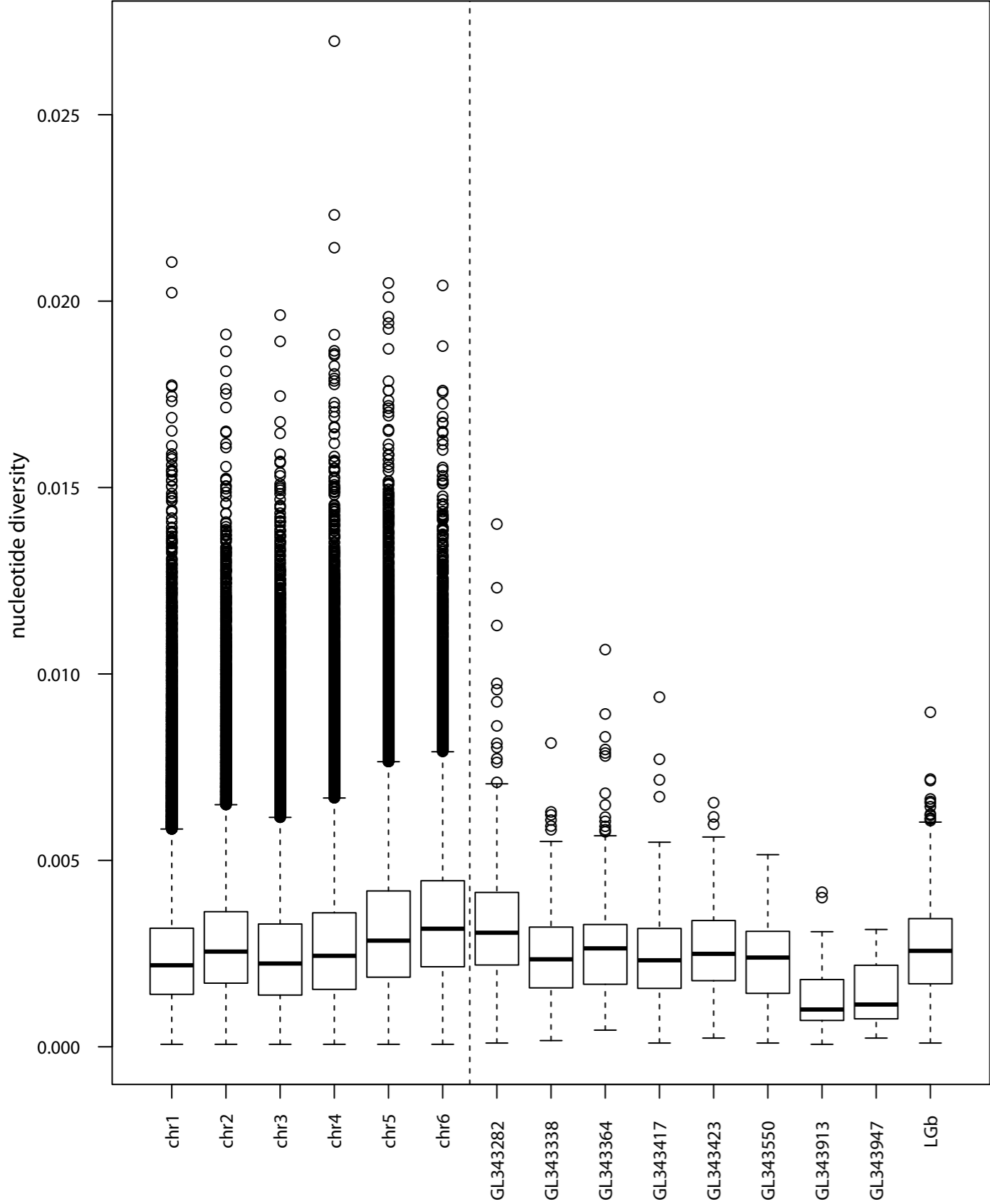

Gulf Atlantic

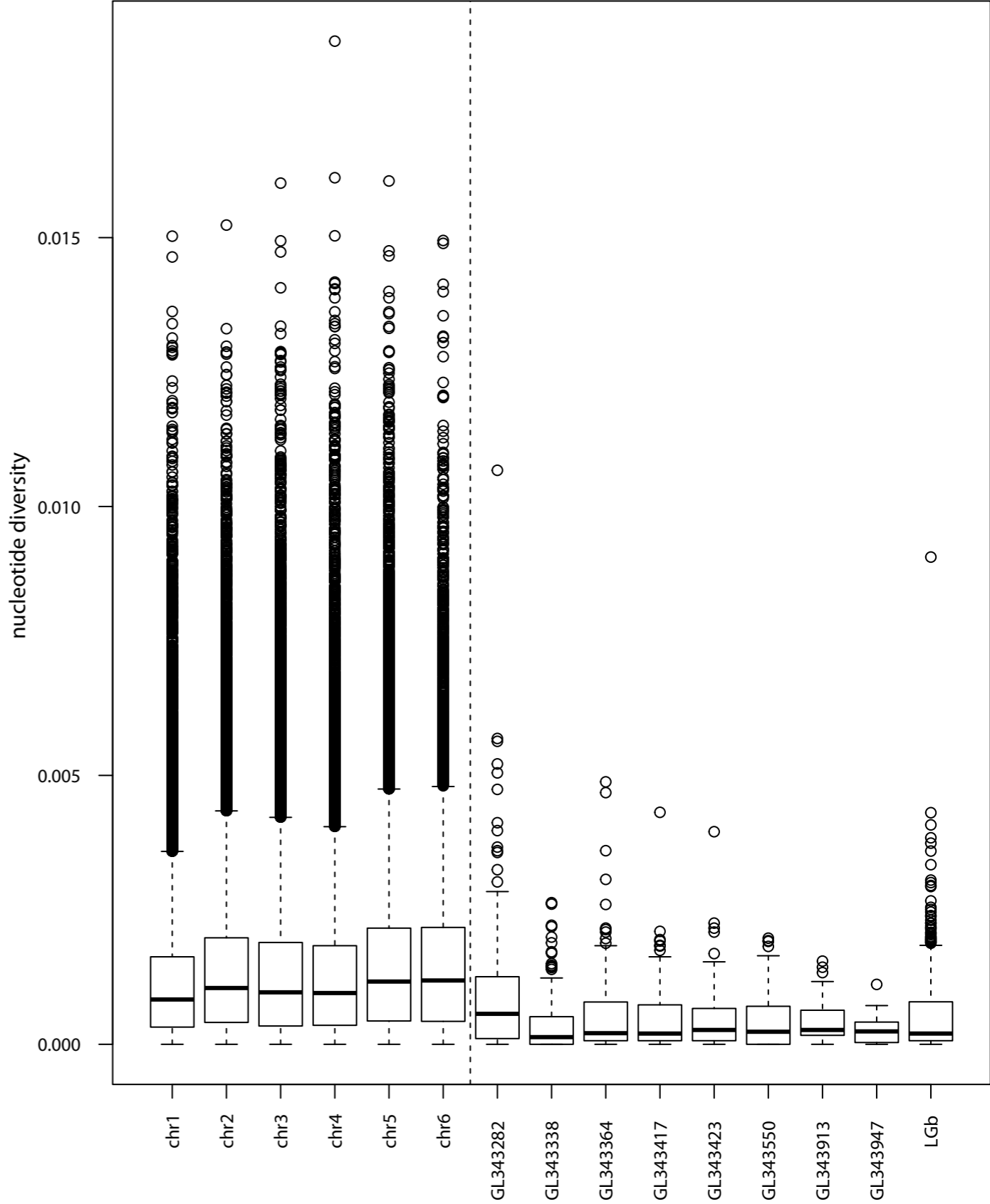

Supplement: evz110_Supplementary_Data [file evz110_supplementary_data.zip › Sup_Fig_4.pdf]
